# Reduction and stabilization of bilirubin with obeticholic acid treatment in patients with primary biliary cholangitis

**DOI:** 10.1111/liv.14429

**Published:** 2020-03-24

**Authors:** Albert Parés, Mitchell Shiffman, Victor Vargas, Pietro Invernizzi, Elizabeth S. Malecha, Alexander Liberman, Leigh MacConell, Gideon Hirschfield

**Affiliations:** ^1^ Hospital Clinic University of Barcelona IDIBAPS CIBERehd Barcelona Spain; ^2^ Liver Institute of Virginia Bon Secours Mercy Health Newport News VA USA; ^3^ Liver Unit Hospital Vall d'Hebron Universitat Autònoma de Barcelona CIBERehd Barcelona Spain; ^4^ Division of Gastroenterology and Center for Autoimmune Liver Diseases San Gerardo Hospital Department of Medicine and Surgery University of Milano‐Bicocca Monza Italy; ^5^ Intercept Pharmaceuticals San Diego CA USA; ^6^ Toronto Centre for Liver Disease Toronto General Hospital and Department of Medicine University of Toronto Toronto Canada

**Keywords:** biliary, bilirubin, cholestasis, cirrhosis

## Abstract

**Background & Aims:**

Total bilirubin is a predictor of survival in primary biliary cholangitis, with the main elevated component being direct bilirubin. The purpose of this post hoc analysis was to assess the efficacy and safety of obeticholic acid across quartiles of varying baseline levels of direct bilirubin in the phase 3, randomized, placebo‐controlled Primary Biliary Cholangitis Obeticholic Acid International Study of Efficacy.

**Methods:**

This analysis assessed patients on the basis of their baseline direct bilirubin level (divided by quartile). Biochemistry and safety outcomes were evaluated within each quartile over time.

**Results:**

In the quartile with the highest baseline direct bilirubin (>5.47 µmol/L), there was a significant reduction in both direct and total bilirubin at Month 12 compared with placebo. Least squares mean (standard error) change from baseline in direct bilirubin at Month 12 was 4.17 (1.42) µmol/L for placebo, −3.48 (1.63) µmol/L for obeticholic acid 5‐10 mg and −3.66 (1.51) µmol/L for obeticholic acid 10 mg (*P* < .0001, obeticholic acid vs placebo); the corresponding values for total bilirubin at Month 12 were 4.38 (1.55) µmol/L for placebo, −4.53 (1.83) µmol/L for obeticholic acid 5‐10 mg and −5.06 (1.64) µmol/L for obeticholic acid 10 mg (*P* < .0001, obeticholic acid vs placebo).

**Conclusions:**

Obeticholic acid treatment was associated with significant reductions in total and direct bilirubin, particularly in patients with high baseline direct bilirubin. Because raised direct bilirubin levels, even within the normal range, are predictive of survival in primary biliary cholangitis, these results suggest substantial benefits of obeticholic acid in at‐risk patients.

**Abbreviations**
ALP: alkaline phosphatase
ALT: alanine aminotransferase
AST: aspartate aminotransferase
GGT: gamma‐glutamyl transferase
LS: least squares
OCA: obeticholic acid
OLE: open‐label extension
PBC: primary biliary cholangitis
POISE: PBC OCA International Study of Efficacy
SE: standard error
UDCA: ursodeoxycholic acid
ULN: upper limit of normal
VAS: visual analog scale

**Key points**

Treatment with obeticholic acid for 12 months improved or stabilized biochemical markers associated with negative long‐term outcomes in patients with primary biliary cholangitis, especially in patients who were considered to be at risk of disease progression.

## 1 INTRODUCTION

Primary biliary cholangitis (PBC) is a chronic autoimmune liver disease characterized by inflammation and the progressive destruction of bile ducts, cholestasis, eventual cirrhosis and death.^1^, ^2^ The prognostic significance of several liver chemistries has been evaluated and found to be associated with disease progression, including alkaline phosphatase (ALP), aspartate aminotransferase (AST) and albumin, with bilirubin being identified as one of the key predictors of survival and prognosis in patients with PBC.^3^, ^4^, ^5^

Rising total bilirubin levels are a late‐stage feature of PBC and are associated with negative long‐term outcomes in patients with the disease.^6^, ^7^ In an analysis of liver chemistries associated with long‐term outcomes in patients with PBC, a total bilirubin level >1x the upper limit of normal (ULN) was a key inverse predictor of transplant‐free survival (hazard ratio: 5.06); 86% of patients with a total bilirubin level ≤1xULN survived for 10 years after enrolment compared with 41% of patients with levels >1xULN.^7^ In addition, it has been found that total bilirubin values, even within the normal range, may have prognostic significance; for example, Trivedi et al showed that the optimum cut‐off value for total bilirubin in predicting liver transplantation or death was 0.70xULN.^5^ The Global PBC Study Group similarly established that rising total bilirubin levels, even within the normal range, are predictive of the 5‐year risk of liver transplantation or death.^8^ In fact, multiple guidelines, response criteria and risk score calculators used for PBC agree that the stabilization or reduction in total bilirubin levels corresponds to reduced risk for disease progression, highlighting the need for PBC treatment options that can stabilize or reduce bilirubin levels in patients with PBC.^4^, ^6^, ^9^, ^10^, ^11^, ^12^, ^13^, ^14^, ^15^, ^16^, ^17^

Most studies and prognostic scores report on only total bilirubin, although a key disease‐related component of total bilirubin is direct or conjugated bilirubin.^6^, ^7^, ^18^, ^19^, ^20^ Tahtaci et al evaluated various haematological and biochemical parameters in patients with advanced or early‐stage PBC and found that, of total and direct bilirubin, only direct bilirubin level was significantly higher in patients with late‐stage disease.^21^ Although fractionation of total bilirubin is not a routine clinical laboratory assessment at this time, the American College of Gastroenterology's clinical guidelines for the evaluation of abnormal liver chemistries recommends that total serum bilirubin levels be separated into direct and indirect fractions.^22^ These guidelines state that direct bilirubin elevations, and not indirect elevations, are often present in cholestatic disorders with impairment in bile flow.^22^ In PBC, the levels of bilirubin are elevated mainly by its direct fraction.^20^, ^23^ Taken together, these data and recommendations suggest that direct bilirubin may be a more sensitive marker for PBC severity than total bilirubin. However, given that total bilirubin is more commonly evaluated, it has emerged as the dominant prognostic marker in various multivariate analyses assessing treatment response and natural history of PBC.

Obeticholic acid (OCA) is a potent and selective farnesoid X receptor agonist that is indicated for PBC as second‐line therapy in combination with ursodeoxycholic acid (UDCA) in adults with an inadequate response to UDCA or as a monotherapy in those unable to tolerate UDCA.^24^, ^25^, ^26^ While the approval of OCA is based largely on reduction in ALP, beneficial effects in bilirubin have also been noted. A phase 2, international, randomized, double‐blind, placebo‐controlled study of OCA as monotherapy showed a decrease from baseline in direct bilirubin levels in patients treated for 3 months with OCA 10 mg and OCA 50 mg compared with placebo.^27^ A phase 2, randomized, double‐blind, placebo‐controlled trial of patients with PBC who had an inadequate response to UDCA also showed decreases in direct bilirubin levels in response to OCA 25 mg and OCA 50 mg compared with placebo.^28^

In the pivotal, phase 3 PBC OCA International Study of Efficacy (POISE) trial, daily doses of OCA 5‐10 mg resulted in a significantly greater number of patients achieving the primary endpoint (ALP<1.67xULN with a ≥15% reduction in ALP from baseline and a total bilirubin level ≤ULN at Month 12) compared with placebo.^25^ Compared with a progressive increase in total bilirubin in the placebo group at Month 12 (2 ± 0.70 μmol/L), patients in the OCA 5 mg titrated to 10 mg (OCA 5‐10 mg) and OCA 10 mg groups experienced a significant reduction in total bilirubin (least squares [LS] mean [standard error, SE]: −0.30 [0.70] and −0.90 [0.70] μmol/L respectively).^25^ OCA treatment therefore prevents increases in bilirubin levels in patients with PBC.

Although total bilirubin is predictive of outcomes, direct bilirubin is the main elevated component in PBC. As such, this post hoc analysis assessed the efficacy and safety of OCA across quartiles of varying baseline levels of direct bilirubin in POISE.

## 2 MATERIALS AND METHODS

### 2.1 Study design

Detailed methods for POISE, including a CONSORT diagram, were previously published (ClinicalTrials.gov, NCT01473524).^25^ Briefly, patients with PBC and an inadequate response to or intolerance of UDCA were randomly assigned to placebo, OCA 5‐10 mg, or OCA 10 mg in the 12‐month, double‐blind, placebo‐controlled, phase 3 study.^25^ The primary composite endpoint in POISE was ALP<1.67xULN with a ≥15% reduction in ALP from baseline and a total bilirubin level ≤ULN at Month 12.^25^ The ULN for total bilirubin was defined as 19.32 μmol/L for women and 25.48 μmol/L for men, and the ULN for direct bilirubin was defined as 3.42 μmol/L for all patients. After the double‐blind phase, patients could enrol in the open‐label extension (OLE) phase, in which all patients were treated with OCA.

In this post hoc analysis, patients in the POISE intent‐to‐treat population (N = 216) were pooled across treatment groups and equally divided into quartiles by baseline direct bilirubin levels (as this is the main elevated component in PBC) to investigate whether there was a relationship between baseline direct bilirubin level and laboratory measures throughout the course of the study.^20^, ^25^ Efficacy and safety analyses were evaluated by direct baseline bilirubin quartiles and treatment groups (Table 1). For comparison with results from direct bilirubin quartiles, efficacy was also evaluated by total bilirubin quartiles. For the assessment of efficacy response rate, discontinuations were considered non‐responders as a conservative form of imputation. Analyses were performed for the double‐blind phase (initial 12 months); long‐term evaluation was performed for the OLE of POISE for up to 36 months of OCA treatment and is presented in the supplemental material. For the OLE analyses, patients were evaluated from the time they initiated OCA treatment; for patients randomly assigned to placebo during the double‐blind phase, the baseline value was the visit immediately prior to OCA initiation.

**TABLE 1:** Baseline direct bilirubin level for each quartile in POISE

Quartile	Direct Bilirubin Cut‐off		Placebo (n)	OCA 5‐10 mg (n)	OCA 10 mg (n)
	(µmol/L)	(mg/dL)			
1	≤2.05	≤0.12	18	20	17
2	2.05 to 3.33	0.12 to 0.20	20	17	18
3	3.33 to 5.47	0.20 to 0.32	17	16	19
4	>5.47	>0.32	18	17	19

Note

Bilirubin values in µmol/L were divided by 17.10 to convert to mg/dL.

Abbreviations: OCA, obeticholic acid; PBC, primary biliary cholangitis; POISE, PBC OCA International Study of Efficacy.

The POISE protocol was approved by local and national ethics and regulatory agencies and was conducted in accordance with Good Clinical Practice guidelines and the Declaration of Helsinki (Seoul, South Korea, October 2008 amendment).^27^ Written informed consent was obtained from each patient included in the study.

### 2.2 Statistical analyses

For the evaluation of the primary endpoint of POISE in each direct bilirubin quartile, *P*‐values for comparing treatments were obtained using the Cochran‐Mantel‐Haenszel general association test stratified by the randomization strata factor. *P*‐values for comparing active treatments vs placebo for direct and total bilirubin, ALP, AST, alanine aminotransferase (ALT) and gamma‐glutamyl transferase (GGT) levels, as well as for the pruritus visual analog scale (VAS) scores, were obtained using an analysis of covariance model with baseline value as a covariate and fixed effects for treatment and the randomization strata factor.

## 3 RESULTS

### 3.1 Baseline characteristics

Baseline values for most demographic characteristics, UDCA use/dose and albumin were generally similar across direct bilirubin quartiles (Table 2). Of the patients who had direct bilirubin levels above the ULN (quartiles 3 and 4), most (83%) had total bilirubin levels within the normal range. The number of male patients and ALP and total bilirubin levels increased progressively by direct bilirubin quartile, whereas platelet count progressively decreased within increasing direct bilirubin quartiles. Baseline values for AST, ALT and GGT did not show clear patterns across quartiles, although the mean value of each of these markers was highest in quartile 4.

**TABLE 2:** Baseline characteristics for direct bilirubin quartiles in POISE

	Quartile 1 (n = 55)	Quartile 2 (n = 55)	Quartile 3 (n = 52)	Quartile 4 (n = 54)
Age, years	57.30 (8.35)	55.40 (10.97)	54.90 (12.03)	55.70 (10.47)
Female, n (%)	54 (98)	52 (95)	47 (90)	43 (80)
Caucasian, n (%)	48 (87)	53 (96)	51 (98)	51 (94)
Duration of PBC, years	7.80 (5.67)	8.80 (6.17)	9.40 (5.58)	8.50 (6.70)
History of PBC‐related pruritus, n (%)	34 (62)	28 (51)	35 (67)	40 (74)
UDCA use, n (%)	54 (98)	49 (89)	47 (90)	50 (93)
UDCA daily dose, mg/kg	16 (4.61)	15.20 (4.25)	16.70 (6.04)	16 (4.47)
ALP, U/L	293.57 (95.03)	312.11 (114.21)	317.66 (88.88)	369.98 (130.11)
AST, U/L	41.07 (13.16)	45.04 (25)	43.83 (15.30)	72.03 (34.08)
ALT, U/L	45.95 (18.89)	53.28 (33.39)	50.52 (24.33)	81.88 (50.25)
GGT, U/L	223.61 (154.46)	210.71 (117.49)	223.35 (164.63)	441.73 (518.85)
Total bilirubin, μmol/L	6.08 (2.58)	7.79 (1.69)	11.55 (3.41)	19.11 (6.97)
>ULN, n (%)	0	0	2 (4)	16 (30)
Direct bilirubin, μmol/L	1.61 (0.15)	2.73 (0.38)	4.23 (0.61)	11.19 (7.05)
>ULN, n (%)	0	0	50 (96)	54 (100)
Platelets, ×10^9^/L	255.19 (83.76)	247.28 (64.75)	234.84 (87.53)	170.81 (75.65)
Albumin, g/dL	4.29 (0.25)	4.40 (0.24)	4.39 (0.28)	4.20 (0.37)

Note

Data are mean (SD) unless otherwise specified. Quartiles were defined by baseline direct bilirubin levels as follows: quartile 1, ≤2.05 µmol/L; quartile 2, 2.05 to 3.33 µmol/L; quartile 3, 3.33 to 5.47 µmol/L; quartile 4, >5.47 µmol/L. The ULN for total bilirubin was as follows: female: 19.32 μmol/L, male: 25.48 μmol/L; the ULN for direct bilirubin was 3.42 μmol/L for all patients.

Abbreviations: ALP, alkaline phosphatase; ALT, alanine aminotransferase; AST, aspartate aminotransferase; GGT, gamma‐glutamyl transferase; POISE, Primary Biliary Cholangitis OCA International Study of Efficacy; UDCA, ursodeoxycholic acid; ULN, upper limit of normal.

### 3.2 Efficacy

**FIGURE 1:**
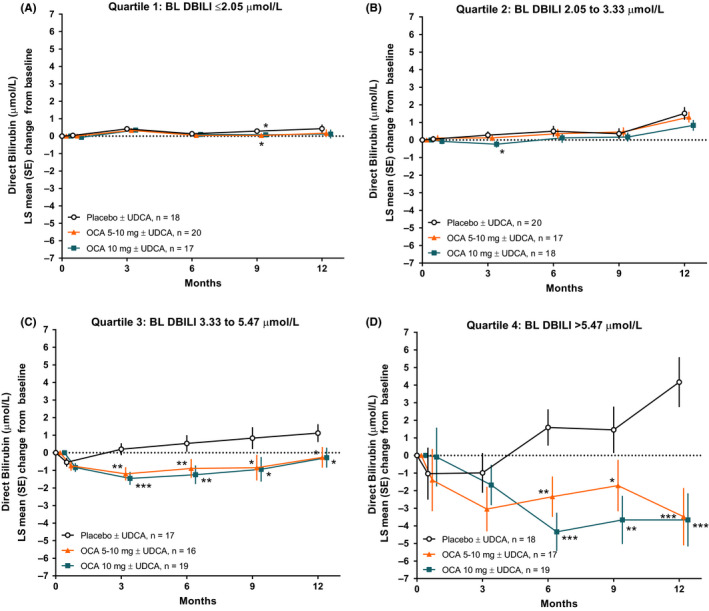
Changes in direct bilirubin in POISE across 12 months of treatment in each direct bilirubin quartile. (A‐B) Quartiles 1 and 2 represent patients with normal baseline direct bilirubin levels. (C‐D) Quartiles 3 and 4 represent patients with baseline direct bilirubin levels generally above the ULN (defined as 3.42 μmol/L). Levels of significance: *P < .05, **P < .01, ***P < .0001; *P*‐values were obtained using an analysis of covariance model. BL, baseline; DBILI, direct bilirubin; OCA, obeticholic acid; PBC, primary biliary cholangitis; POISE, PBC OCA International Study of Efficacy; UDCA, ursodeoxycholic acid; ULN, upper limit of normal

In a comparison between quartiles, direct bilirubin levels remained stable in quartiles 1 and 2 across all treatment groups (Figure 1A,B). In contrast, both dosages of OCA resulted in a reduction in direct bilirubin levels in quartiles 3 and 4 after 12 months of randomized treatment, whereas direct bilirubin levels increased in the placebo group (Figure 1C,D). Comparison between treatment groups within quartiles 3 and 4 showed a statistically significant difference in LS mean change from baseline in direct bilirubin levels at Month 12 relative to placebo (quartile 3: OCA 5‐10 mg, −0.26 µmol/L; OCA 10 mg, −0.28 µmol/L; placebo, 1.12 µmol/L, both OCA groups, *P* < .05; quartile 4: OCA 5‐10 mg, −3.48 µmol/L; OCA 10 mg, −3.66 µmol/L; placebo, 4.17 µmol/L, both OCA groups, *P* < .0001). Long‐term assessment throughout 36 months of the OLE also showed the most pronounced decrease in direct bilirubin levels in direct bilirubin quartiles 3 and 4 (Figure S1).

**FIGURE 2:**
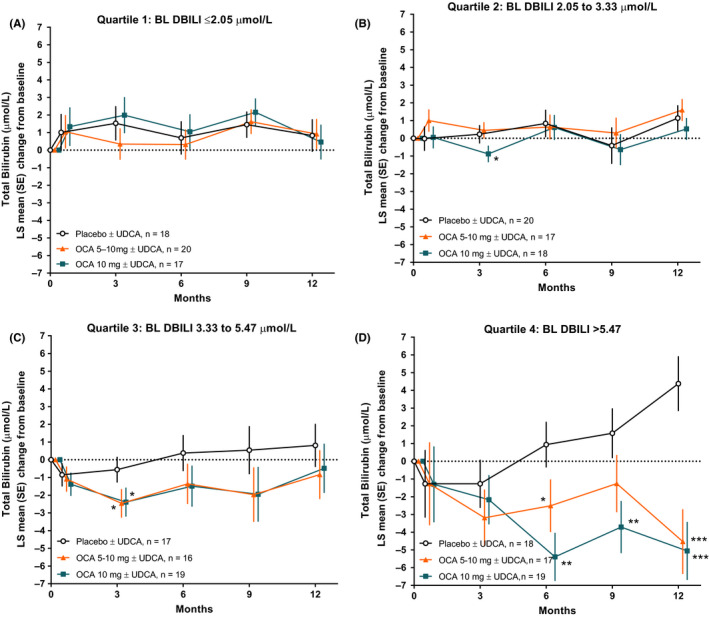
Changes in total bilirubin in POISE across 12 months of treatment in each direct bilirubin quartile. (A‐B) Quartiles 1 and 2 represent patients with normal baseline direct bilirubin levels. (C‐D) Quartiles 3 and 4 represent patients with baseline direct bilirubin levels generally above the ULN (defined as 3.42 μmol/L). Levels of significance: *P < .05, **P < .01, ***P < .0001; *P*‐values were obtained using an analysis of covariance model. BL, baseline; DBILI, direct bilirubin; OCA, obeticholic acid; POISE, Primary Biliary Cholangitis OCA International Study of Efficacy; UDCA, ursodeoxycholic acid; ULN, upper limit of normal

When total bilirubin was compared across direct bilirubin quartiles, total bilirubin levels remained relatively stable in quartiles 1 and 2 (Figure 2A,B). In contrast, both dosages of OCA resulted in reduction of total bilirubin levels in quartiles 3 and 4 after 12 months of treatment, whereas total bilirubin levels steadily increased from Months 3 to 12 in the placebo group (Figure 2C,D). The LS mean change in total bilirubin levels from baseline at Month 12 was statistically significant for both dosage groups of OCA in direct bilirubin quartile 4 relative to placebo (OCA 5‐10 mg, −4.53 µmol/L; OCA 10 mg, −5.06 µmol/L; placebo, 4.38 µmol/L, both OCA groups, *P* < .0001). There was a non‐significant reduction in total bilirubin from baseline at Month 12 in quartile 3 (OCA 5‐10 mg, −0.84 µmol/L; OCA 10 mg, −0.48 µmol/L; placebo, 0.81 µmol/L). Long‐term assessment throughout 36 months of the OLE showed sustained reductions in total bilirubin levels for direct bilirubin quartiles 3 and 4 (Figure S2).

Additional analyses of patients in the double‐blind phase showed results similar to those of direct bilirubin when assessed by baseline total bilirubin quartiles (Figure S3) or total bilirubin >0.70xULN, a cut‐off value for bilirubin in predicting liver transplantation or death (Figure S4).^5^, ^7^

**FIGURE 3:**
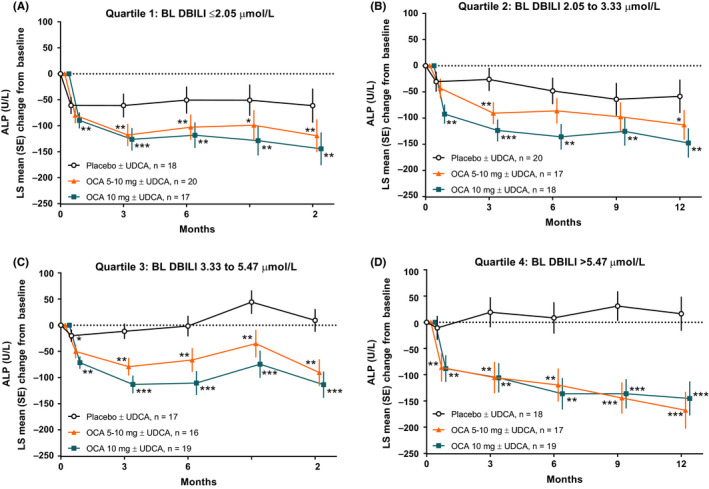
Changes in ALP in POISE across 12 months of treatment in each direct bilirubin quartile. (A‐B) Quartiles 1 and 2 represent patients with normal baseline direct bilirubin levels. (C‐D) Quartiles 3 and 4 represent patients with baseline direct bilirubin levels generally above the ULN (defined as 3.42 μmol/L). Levels of significance: *P < .05, **P < .01, ***P < .0001; *P*‐values were obtained using an analysis of covariance model. ALP, alkaline phosphatase; BL, baseline; DBILI, direct bilirubin; OCA, obeticholic acid; POISE, Primary Biliary Cholangitis OCA International Study of Efficacy; UDCA, ursodeoxycholic acid; ULN, upper limit of normal

Over 12 months of randomized treatment, both dosages of OCA resulted in statistically significant reductions in ALP levels across each direct bilirubin quartile relative to placebo (Figure 3). The LS mean change in ALP from baseline at Month 12 for OCA groups relative to placebo was statistically significant for each quartile (all quartiles, *P* < .05 for both OCA groups vs placebo). The magnitude of the LS mean change in ALP was generally similar for each OCA dose across quartiles. Long‐term assessment throughout 36 months of the OLE also showed reductions in ALP levels for each direct bilirubin quartile (Figure S5).

Compared with patients randomly assigned to placebo, more patients randomly assigned to OCA achieved the primary endpoint of POISE for each baseline direct bilirubin quartile at Month 12, an effect that was significant for OCA 10 mg in quartile 1, both doses of OCA in quartiles 2 and 3 and OCA 5‐10 mg in quartile 4, and this was generally consistent with or greater than the effect in the overall cohort (Figure S6). In a long‐term assessment of open‐label treatment, patients in direct bilirubin quartile 4 who received either dose of OCA in the double‐blind portion of the study had higher responder rates at 12, 24 and 36 months than patients who had received placebo in the double‐blind phase; the other quartiles did not show clear differences or patterns across groups (Figure S7A‐C).

In a comparison of all baseline direct bilirubin quartiles, ALT, GGT and AST levels were also reduced throughout 12 months of treatment in both OCA dosage groups vs placebo, and within each quartile, this effect was generally significant throughout each time point for each marker (Figure S8‐10). In addition, 12 months of treatment with OCA reduced the median estimated risk of death or liver transplantation at years 5, 10 and 15 per the GLOBE score and UK‐PBC risk score (Figure S11‐12). The largest reduction in estimated risk was observed in quartile 4, where comparison between OCA and placebo achieved statistical significance (*P* < .01) for both OCA dosage groups across all time points.

### 3.3 Safety and tolerability

In patients receiving OCA 5‐10 mg during the double‐blind phase, similar incidences of pruritus were observed in each baseline direct bilirubin quartile, and they remained generally similar to placebo in quartiles 1 and 2; however, the incidence of pruritus generally increased with increasing quartiles in the OCA 10 mg group compared with placebo (Table S1). One death unrelated to OCA occurred in a patient receiving OCA 5‐10 mg; the patient was in direct bilirubin quartile 4. During the OLE, the exposure‐adjusted treatment‐emergent adverse event rate for the total OCA group generally increased with increasing direct bilirubin quartile for overall adverse events, pruritus, serious adverse events and hepatobiliary disorders (Table S2). Hyperbilirubinemia was reported in 1 patient during the OLE in quartile 4 (data not shown). One death occurred in the OLE that was deemed unrelated to OCA.^29^

In direct bilirubin quartiles 1 and 2, there were no statistical differences between treatment groups in pruritus VAS scores; pruritus VAS scores were statistically higher in the OCA 10 mg group relative to placebo during the first 3‐6 months of OCA treatment in direct bilirubin quartiles 3 and 4 (Figure S13). The median change from baseline in pruritus VAS scores for all treatment groups was ≤1 at all time points in the double‐blind phase in quartiles 1 and 2 (data not shown). After 36 months of the OLE, a general increase in pruritus VAS scores was observed in direct bilirubin quartiles 3 and 4 (Figure S14).

## 4 DISCUSSION

### 4.1 Summary of the results and their implications

These results suggest that high baseline direct as well as total bilirubin levels identify the risk for worsening of biochemistry in patients with PBC. Patients receiving placebo with baseline direct bilirubin >ULN (quartiles 3 and 4) showed evidence of disease progression over the 12‐month course of the POISE placebo‐controlled study, as indicated by rising direct 12‐month bilirubin levels. Treatment with OCA for 12 months led to significant improvements in direct and total bilirubin levels in quartile 4 compared with placebo and prevented a rise in ALP, AST, ALT and GGT liver chemistries across each baseline direct bilirubin quartile. Significant reductions in the estimated risk of negative outcomes per the GLOBE score and UK‐PBC risk score at 5‐, 10‐ and 15‐year time points were also observed in quartile 4 compared with placebo. The results of this study further support the efficacy of OCA, with observed general reductions from baseline in each of these parameters regardless of disease severity.

Although total bilirubin is commonly reported as a prognostic measure for PBC, data from this study support greater exploration of direct bilirubin levels as a measure of disease progression.^20^ The majority of patients in quartiles 3 and 4 had total bilirubin levels within the normal range (70% of patients in quartile 4), but their direct bilirubin levels were above the ULN (mean direct bilirubin level in quartile 4 was 3.27xULN). These patients showed a decreasing trend in platelet count and an increasing trend in ALP level at baseline, corresponding with increasing total and direct bilirubin levels between quartiles. Analyses of quartiles defined by baseline total bilirubin levels showed results similar to those of direct bilirubin quartiles, and a clear increasing trend was observed in the placebo group for direct bilirubin more so than total bilirubin in quartile 3, further supporting the possible role of direct bilirubin as a more sensitive measure of disease severity. These data combined suggest that direct bilirubin measurements should be considered in a clinical setting when a patient presents with increasing total bilirubin levels, even if the increase in total bilirubin is considered to be within the normal range.

Regarding safety, the incidence of treatment‐emergent pruritus in patients receiving OCA 5‐10 mg was similar to that of placebo in the lower baseline direct bilirubin quartiles (quartiles 1 and 2), whereas patients in quartile 4 (with the highest baseline direct bilirubin levels) showed an increased incidence of pruritus‐related discontinuations and the largest increase in pruritus severity relative to other quartiles, as assessed by the VAS. This group of patients also had the greatest benefit from OCA treatment per biochemical evaluations. It should be noted that all patients participating in the POISE study had a high risk of disease progression because of an inadequate response to or intolerance of UDCA; however, the baseline disease severity for all patients was generally mild to moderate, with few patients having compensated cirrhosis (Child‐Pugh A cirrhosis).^25^ Incorrect doses of OCA can pose a risk to patients with moderate to severe hepatic impairment (Child‐Pugh B/C cirrhosis), and it is important to follow recommended dosing and administration provisions with OCA.^24^

Evaluation of treatment with OCA in patients with advanced PBC continues in two ongoing phase 4 clinical studies. The Study of Obeticholic Acid Evaluating Clinical Outcomes in Patients With Primary Biliary Cholangitis (COBALT, study 747‐302; EudraCT, 2014‐005012‐42; ClinicalTrials.gov, NCT02308111) is assessing the long‐term clinical outcomes of OCA, and study 747‐401 (EudraCT, 2017‐001762‐13; ClinicalTrials.gov, NCT03633227) is evaluating the pharmacokinetics and safety of OCA in patients with PBC and moderate to severe hepatic impairment (Child‐Pugh B and Child‐Pugh C cirrhosis).^30^, ^31^

### 4.2 Literature context

In patients with PBC, the development of hyperbilirubinemia is associated with disease progression and impaired bilirubin excretion, and the elevation of direct bilirubin may result from obstructed bile flow, altered bile ductular integrity or reduced production of bile because of defective activity of bile efflux transporters.^20^, ^32^ Results from this study support previous data showing that OCA may improve total and direct bilirubin levels^25^; however, the novelty of the analyses performed in this study resides in the in‐depth evaluation of the effect of OCA across various baseline direct and total bilirubin levels. The demonstrated benefits of OCA in the analyses shown here are expected to be clinically significant, as UDCA‐treated patients with PBC who had higher total bilirubin levels, even within the normal range, were shown to have an increased risk of liver transplantation or death compared with patients with lower total bilirubin levels.^5^, ^8^, ^33^ Our finding that OCA reduces both total and direct bilirubin in patients with elevated direct bilirubin (quartile 4) supports the potential for OCA to delay the occurrence of outcome events, as a bilirubin level of 1.60xULN is associated with an accelerated increase in bilirubin and a median time of 19 months to an outcome event.^34^ Even with total bilirubin levels well within the normal range, the data presented here indicate that direct bilirubin levels may be significantly elevated. Although many guidelines and established risk criteria include total bilirubin, lower thresholds within the normal range may need to be considered for clinical use.^4^, ^6^, ^9^, ^10^, ^11^, ^12^, ^13^, ^14^, ^15^, ^16^, ^17^ This is further exemplified by the inclusion of total bilirubin as a continuous variable within the UK‐PBC risk score and Global PBC risk score, suggesting that lower total bilirubin levels within any range contribute to a lower risk of liver‐related/all‐cause mortality and liver transplantation.^9^, ^16^ These findings support the use of direct bilirubin measurements as a potentially more distinct marker of disease progression, which may shape the standards currently used in the field. However, in clinical practice, direct bilirubin levels are typically measured only when total bilirubin level is above the ULN, a fact that may attenuate this proposal.

### 4.3 Limitations of the study

The analyses performed here are post hoc; therefore, this study was not specifically powered to detect differences within quartiles. In addition, data presented from the OLE did not include a placebo group for comparison to account for natural disease progression. The study population was not representative of one with a high incidence of jaundice; jaundice occurs at total bilirubin levels above 51.30 μmol/L, and the highest total bilirubin quartile in POISE showed a mean level of 19.12 μmol/L.^35^

### 4.4 Strengths of the study

The analyses presented here support the benefits of OCA for various PBC liver chemistries, including total and direct bilirubin levels, even in at‐risk patient populations. The data used for these analyses were collected prospectively as part of an international, randomized, placebo‐controlled, phase 3 study.

## CONFLICTS OF INTEREST

AP has received grant funding, personal fees and advisory board fees from Intercept Pharmaceuticals; advisory board fees and personal fees from Novartis; and personal fees from CymaBay Therapeutics and Inova Diagnostics. MLS has received grant funding and consulting, advisory board and speaker fees from Intercept Pharmaceuticals; grant funding and consulting and speaker fees from Gilead Sciences; grant funding and speaker fees from Enanata Pharmaceuticals; grant funding from CymaBay Therapeutics, Genkyotex, Afimmune, Conatus Pharmaceuticals, Exalenz Bioscience, Genfit, NGM Biopharmaceuticals, Novartis and Shire; grant funding and personal fees from AbbVie, Dova Pharmaceuticals and Valeant Pharmaceuticals; and personal fees from Bayer, Bristol Myers‐Squibb, Easai, Daiichi Sankyo, Mallinckrodt, Merck, Optum RX and Shionogi. VV has received speaking fees from Intercept Pharmaceuticals. PI has received grant funding and advisory board fees from Intercept Pharmaceuticals; grant funding from Bruschettini and Gilead Sciences; grant funding and consultant fees from Menarini Diagnostics; consultant fees from Genkyotex and International Laboratory; and is co‐owner of LIVERA. GMH has received personal fees from Intercept Pharmaceuticals, CymaBay Therapeutics and GSK; and grant funding from Gilead Sciences and Faulk Pharmaceuticals. ESM, AL and LM are employees of and stock shareholders for Intercept Pharmaceuticals.

## ETHICAL APPROVAL STATEMENT

The POISE protocol was approved by local and national ethics and regulatory agencies and was conducted in accordance with Good Clinical Practice guidelines and the Declaration of Helsinki (Seoul, South Korea, October 2008 amendment).

## PATIENT CONSENT STATEMENT

Written informed consent was obtained from each patient included in the study.

## Supporting information

Supplementary Material

## References

[1] Hirschfield GM , Gershwin ME . The immunobiology and pathophysiology of primary biliary cirrhosis. Annu Rev Pathol. 2013;8:303‐330. 23347352 10.1146/annurev-pathol-020712-164014

[2] Lindor KD , Bowlus CL , Boyer J , Levy C , Mayo M . Primary biliary cholangitis: 2018 practice guidance from the American Association for the Study of Liver Diseases. Hepatology. 2019;69(1):394‐419. 30070375 10.1002/hep.30145

[3] Kouroumalis E , Samonakis D , Voumvouraki A . Biomarkers for primary biliary cholangitis: current perspectives. Hepat Med. 2018;10:43‐53. 29950909 10.2147/HMER.S135337 PMC6014437

[4] Lindor KD , Gershwin ME , Poupon R , Kaplan M , Bergasa NV , Heathcote EJ . Primary biliary cirrhosis. Hepatology. 2009;50(1):291‐308. 19554543 10.1002/hep.22906

[5] Trivedi PJ , Bruns T , Cheung A , et al. Optimising risk stratification in primary biliary cirrhosis: AST/platelet ratio index predicts outcome independent of ursodeoxycholic acid response. J Hepatol. 2014;60(6):1249‐1258. 24548531 10.1016/j.jhep.2014.01.029

[6] European Association for the Study of the Liver . EASL Clinical Practice Guidelines: the diagnosis and management of patients with primary biliary cholangitis. J Hepatol. 2017;67(1):145‐172. 28427765 10.1016/j.jhep.2017.03.022

[7] Lammers WJ , van Buuren HR , Hirschfield GM , et al. Levels of alkaline phosphatase and bilirubin are surrogate end points of outcomes of patients with primary biliary cirrhosis: an international follow‐up study. Gastroenterology. 2014;147(6):1338‐1349.e1335; quiz e1315. 25160979 10.1053/j.gastro.2014.08.029

[8] Murillo Perez CF , Gulamhusein A , van Buuren HR , et al. Bilirubin is predictive of transplant‐free survival even within the normal range in primary biliary cholangitis patients. Hepatology. 2017;66:41A‐42A.

[9] Carbone M , Sharp SJ , Flack S , et al. The UK‐PBC risk scores: derivation and validation of a scoring system for long‐term prediction of end‐stage liver disease in primary biliary cholangitis. Hepatology. 2016;63(3):930‐950. 26223498 10.1002/hep.28017 PMC6984963

[10] Corpechot C , Abenavoli L , Rabahi N , et al. Biochemical response to ursodeoxycholic acid and long‐term prognosis in primary biliary cirrhosis. Hepatology. 2008;48(3):871‐877. 18752324 10.1002/hep.22428

[11] Corpechot C , Chazouilleres O , Poupon R . Early primary biliary cirrhosis: biochemical response to treatment and prediction of long‐term outcome. J Hepatol. 2011;55(6):1361‐1367. 21703194 10.1016/j.jhep.2011.02.031

[12] Freeman RB Jr , Wiesner RH , Harper A , et al. The new liver allocation system: moving toward evidence‐based transplantation policy. Liver Transpl. 2002;8(9):851‐858. 12200791 10.1053/jlts.2002.35927

[13] Hirschfield GM , Dyson JK , Alexander GJM , et al. The British Society of Gastroenterology/UK‐PBC primary biliary cholangitis treatment and management guidelines. Gut. 2018;67(9):1568‐1594. 29593060 10.1136/gutjnl-2017-315259 PMC6109281

[14] Kilmurry MR , Heathcote EJ , Cauch‐Dudek K , et al. Is the Mayo model for predicting survival useful after the introduction of ursodeoxycholic acid treatment for primary biliary cirrhosis? Hepatology. 1996;23(5):1148‐1153. 8621147 10.1002/hep.510230532

[15] Kuiper EMM , Hansen BE , de Vries RA , et al. Improved prognosis of patients with primary biliary cirrhosis that have a biochemical response to ursodeoxycholic acid. Gastroenterology. 2009;136(4):1281‐1287. 19208346 10.1053/j.gastro.2009.01.003

[16] Lammers WJ , Hirschfield GM , Corpechot C , et al. Development and validation of a scoring system to predict outcomes of patients with primary biliary cirrhosis receiving ursodeoxycholic acid therapy. Gastroenterology. 2015;149(7):1804‐1812.e1804. 26261009 10.1053/j.gastro.2015.07.061

[17] Mayo MJ , Parkes J , Adams‐Huet B , et al. Prediction of clinical outcomes in primary biliary cirrhosis by serum enhanced liver fibrosis assay. Hepatology. 2008;48(5):1549‐1557. 18846542 10.1002/hep.22517 PMC2597274

[18] Bowlus CL . Obeticholic acid for the treatment of primary biliary cholangitis in adult patients: clinical utility and patient selection. Hepat Med. 2016;8:89‐95. 27621676 10.2147/HMER.S91709 PMC5012622

[19] Jung HE , Jang JY , Jeong SW , et al. Prognostic indicators in primary biliary cirrhosis: significance of revised IAHG (International Autoimmune Hepatitis Group) score. Clin Mol Hepatol. 2012;18(4):375‐382. 23323253 10.3350/cmh.2012.18.4.375 PMC3540374

[20] Reshetnyak VI . Primary biliary cirrhosis: clinical and laboratory criteria for its diagnosis. World J Gastroenterol. 2015;21(25):7683‐7708. 26167070 10.3748/wjg.v21.i25.7683 PMC4491957

[21] Tahtaci M , Yurekli OT , Bolat AD , et al. Increased mean platelet volume is related to histologic severity of primary biliary cirrhosis. Eur J Gastroenterol Hepatol. 2015;27(12):1382‐1385. 26308712 10.1097/MEG.0000000000000463 PMC4892760

[22] Kwo PY , Cohen SM , Lim JK . ACG clinical guideline: evaluation of abnormal liver chemistries. Am J Gastroenterol. 2017;112(1):18‐35. 27995906 10.1038/ajg.2016.517

[23] Wang H , Xu H , Wang X , et al. Red blood cell distribution width to platelet ratio is related to histologic severity of primary biliary cirrhosis. Medicine (Baltimore). 2016;95(11):e3114. 26986159 10.1097/MD.0000000000003114 PMC4839940

[24] Intercept Pharmaceuticals . OCALIVA [package insert]. New York, NY: Intercept Pharmaceuticals; 2018.

[25] Nevens F , Andreone P , Mazzella G , et al. A placebo‐controlled trial of obeticholic acid in primary biliary cholangitis. N Engl J Med. 2016;375(7):631‐643. 27532829 10.1056/NEJMoa1509840

[26] Pellicciari R , Fiorucci S , Camaioni E , et al. 6alpha‐ethyl‐chenodeoxycholic acid (6‐ECDCA), a potent and selective FXR agonist endowed with anticholestatic activity. J Med Chem. 2002;45(17):3569‐3572. 12166927 10.1021/jm025529g

[27] Kowdley KV , Luketic V , Chapman R , et al. A randomized trial of obeticholic acid monotherapy in patients with primary biliary cholangitis. Hepatology. 2018;67(5):1890‐1902. 29023915 10.1002/hep.29569 PMC5947631

[28] Hirschfield GM , Mason A , Luketic V , et al. Efficacy of obeticholic acid in patients with primary biliary cirrhosis and inadequate response to ursodeoxycholic acid. Gastroenterology. 2015;148(4):751‐761.e758. 25500425 10.1053/j.gastro.2014.12.005

[29] Trauner M , Nevens F , Shiffman ML , et al. Long‐term efficacy and safety of obeticholic acid for patients with primary biliary cholangitis: 3‐year results of an international open‐label extension study. Lancet Gastroenterol Hepatol. 2019;4(6):445‐453. 30922873 10.1016/S2468-1253(19)30094-9

[30] Clinicaltrials.gov . Phase 4 study of obeticholic acid evaluating clinical outcomes in patients with primary biliary cholangitis (COBALT). 2019 https://clinicaltrials.gov/ct2/show/NCT02308111. Accessed February 20, 2019

[31] Clinicaltrials.gov . Study of OCA evaluating pharmacokinetics and safety in patients with PBC and hepatic impairment. 2019 https://clinicaltrials.gov/ct2/show/NCT03633227. Accessed February 20, 2019.

[32] Levitt DG , Levitt MD . Quantitative assessment of the multiple processes responsible for bilirubin homeostasis in health and disease. Clin Exp Gastroenterol. 2014;7:307‐328. 25214800 10.2147/CEG.S64283 PMC4159128

[33] Gordon S , Rodriguez C , Romanelli R , Haller I . Serum bilirubin within normal range is associated with an increasing risk of mortality in patients with primary biliary cholangitis regardless of ursodeoxycholic acid treatment. Hepatology. 2018;68(1):31A.

[34] Harms MH , Pares A , Mason AL , et al. Behavioral patterns of total serum bilirubin prior to major clinical endpoints in 3529 patients with primary biliary cholangitis. J Hepatol. 2016;64(2):S633‐S634.

[35] Fargo MV , Grogan SP , Saguil A . Evaluation of jaundice in adults. Am Fam Physician. 2017;95(3):164‐168. 28145671

