# Supplementary material for: Reduction and stabilization of bilirubin with obeticholic acid treatment in patients with primary biliary cholangitis
Source: Liver Int. 2020 Mar 24;40(5):1121–9. doi: 10.1111/liv.14429 (PMC7317821; doi:10.1111/liv.14429)
Supplement: Supplementary file 1 — Supplementary Material [file LIV-40-1121-s001.doc]

**Supplementary Material**

This supplement provides additional information about the results described and referenced in the main manuscript.

Supplement to: Parés A, Shiffman M, Vargas V, Invernizzi P, Malecha ES, Liberman A, et al. Reduction and Stabilization of Bilirubin With Obeticholic Acid Treatment in Patients With Primary Biliary Cholangitis

**Table of Contents**

Supplemental Figures 4

**Fig. S1. Changes in direct bilirubin in the POISE OLE across 36 months of treatment in each direct bilirubin quartile by double-blind treatment in the safety population** 4

**Fig. S2. Changes in total bilirubin in the POISE OLE across 36 months of treatment in each direct bilirubin quartile by double-blind treatment in the safety population** 5

**Fig. S3. Changes in total bilirubin in POISE across 12 months of treatment in each total bilirubin quartile** 6

**Fig. S4. Changes in total bilirubin across 12 months of treatment in the POISE double-blind phase in patients with total bilirubin ≤0.70xULN or >0.70xULN at baseline** 7

**Fig. S5. Changes in ALP in the POISE OLE across 36 months of treatment in each direct bilirubin quartile by double-blind treatment in the safety population** 8

**Fig. S6. Primary endpoint of POISE at 12 months of treatment in each direct bilirubin quartile in the ITT population (N=216)** 9

**Fig. S7. Primary endpoint of POISE by double-blind treatment in the safety population in the OLE (N=193)** 10

**Fig. S8. Changes in ALT in POISE across 12 months of treatment in each direct bilirubin quartile** 11

**Fig. S9. Changes in GGT in POISE across 12 months of treatment in each direct bilirubin quartile** 12

**Fig. S10. Changes in AST in POISE across 12 months of treatment in each direct bilirubin quartile** 13

**Fig. S11. Changes in estimated risk using the GLOBE score in POISE at 12 months of treatment in each direct bilirubin quartile** 14

**Fig. S12. Changes in estimated risk using the UK-PBC risk score in POISE at 12 months of treatment in each direct bilirubin quartile** 15

**Fig. S13. Changes in pruritus VAS scores in POISE across 12 months of treatment in each direct bilirubin quartile** 16

**Fig. S14. Changes in pruritus VAS scores in the POISE OLE across 36 months of treatment in each direct bilirubin quartile by double-blind treatment in the safety population** 17

Supplemental Tables 18

**Table S1. Treatment-emergent adverse events by baseline direct bilirubin quartile in POISE in the safety population** 18

**Table S2. Exposure-adjusted treatment-emergent adverse event rate by baseline direct bilirubin quartile in the POISE OLE in the safety population** 19-20

Supplemental Figures
**
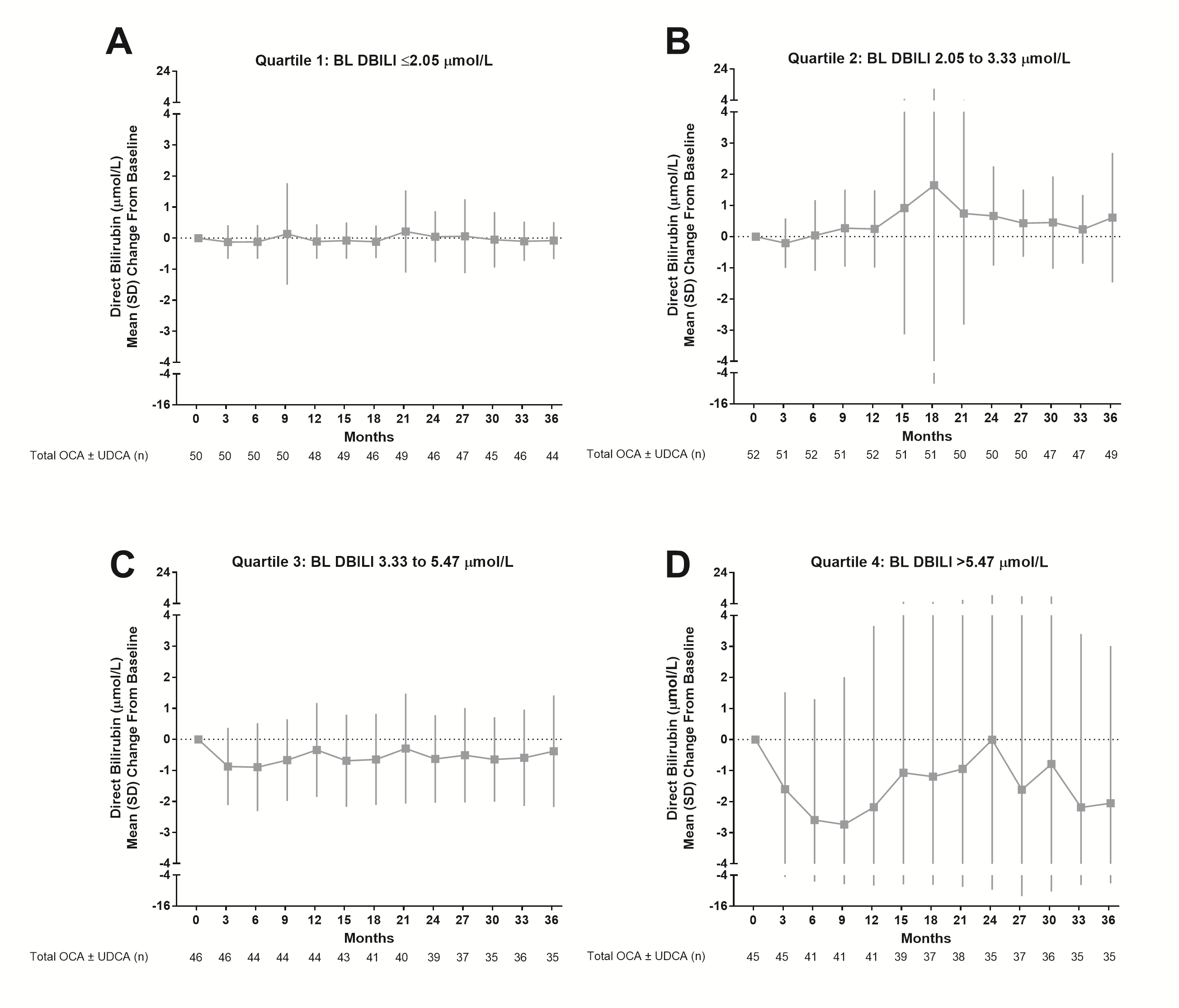
** Fig. S1. Changes in direct bilirubin in the POISE OLE across 36 months of treatment in each direct bilirubin quartile by double-blind treatment in the safety population. **(QC’ed by MS-- blue highlights-- t425.2-efflabs-dbiliq-si-oca-m36-09NOV2018)**

Patients enrolled in the POISE OLE were divided into quartiles by baseline direct bilirubin levels. (A-B) Quartiles 1 and 2 represent patients with normal baseline direct bilirubin levels. (C-D) Quartiles 3 and 4 represent patients with baseline direct bilirubin levels generally above the ULN (defined as 3.42 micromol/L).

Abbreviations: BL, baseline; DBILI, direct bilirubin; OCA, obeticholic acid; OLE, open-label extension; POISE, Primary biliary cholangitis OCA International Study of Efficacy; SD, standard deviation; UDCA, ursodeoxycholic acid; ULN, upper limit of normal.

**(QC’ed by MS-- green highlights-- t425.2-efflabs-dbiliq-si-oca-m36-09NOV2018)**


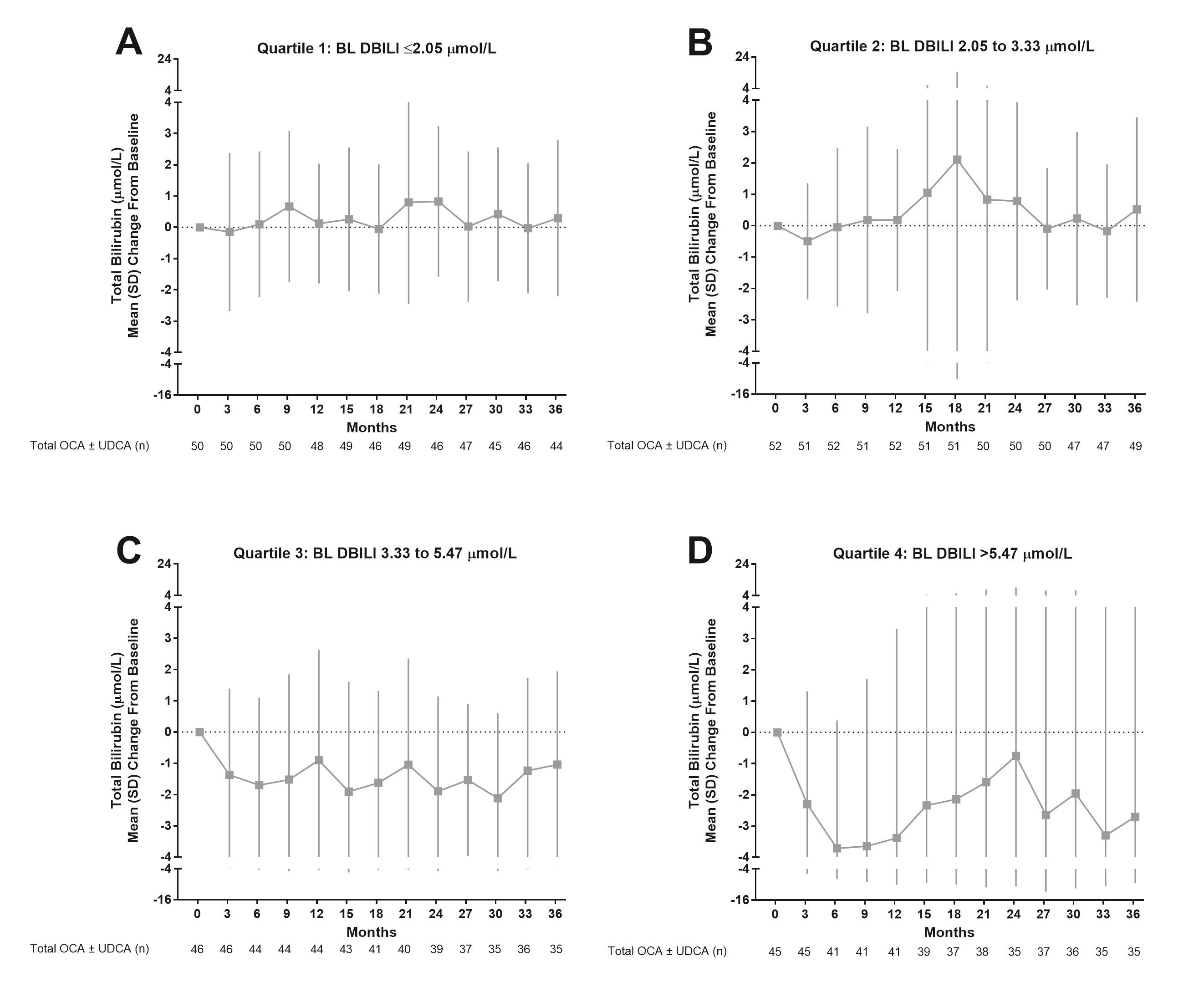
Fig. S2. Changes in total bilirubin in the POISE OLE across 36 months of treatment in each direct bilirubin quartile by double-blind treatment in the safety population. Patients enrolled in the POISE OLE were divided into quartiles by baseline direct bilirubin levels. (A-B) Quartiles 1 and 2 represent patients with normal baseline direct bilirubin levels. (C-D) Quartiles 3 and 4 represent patients with baseline direct bilirubin levels generally above the ULN (defined as 3.42 micromol/L).

Abbreviations: BL, baseline; DBILI, direct bilirubin; OCA, obeticholic acid; OLE, open-label extension; PBC, primary biliary cholangitis; POISE, PBC OCA International Study of Efficacy; SD, standard deviation; UDCA, ursodeoxycholic acid ULN, upper limit of normal.

**(t355.3.2‐efflabs‐tbiliq‐si‐hrm‐db301‐13APR2017)**


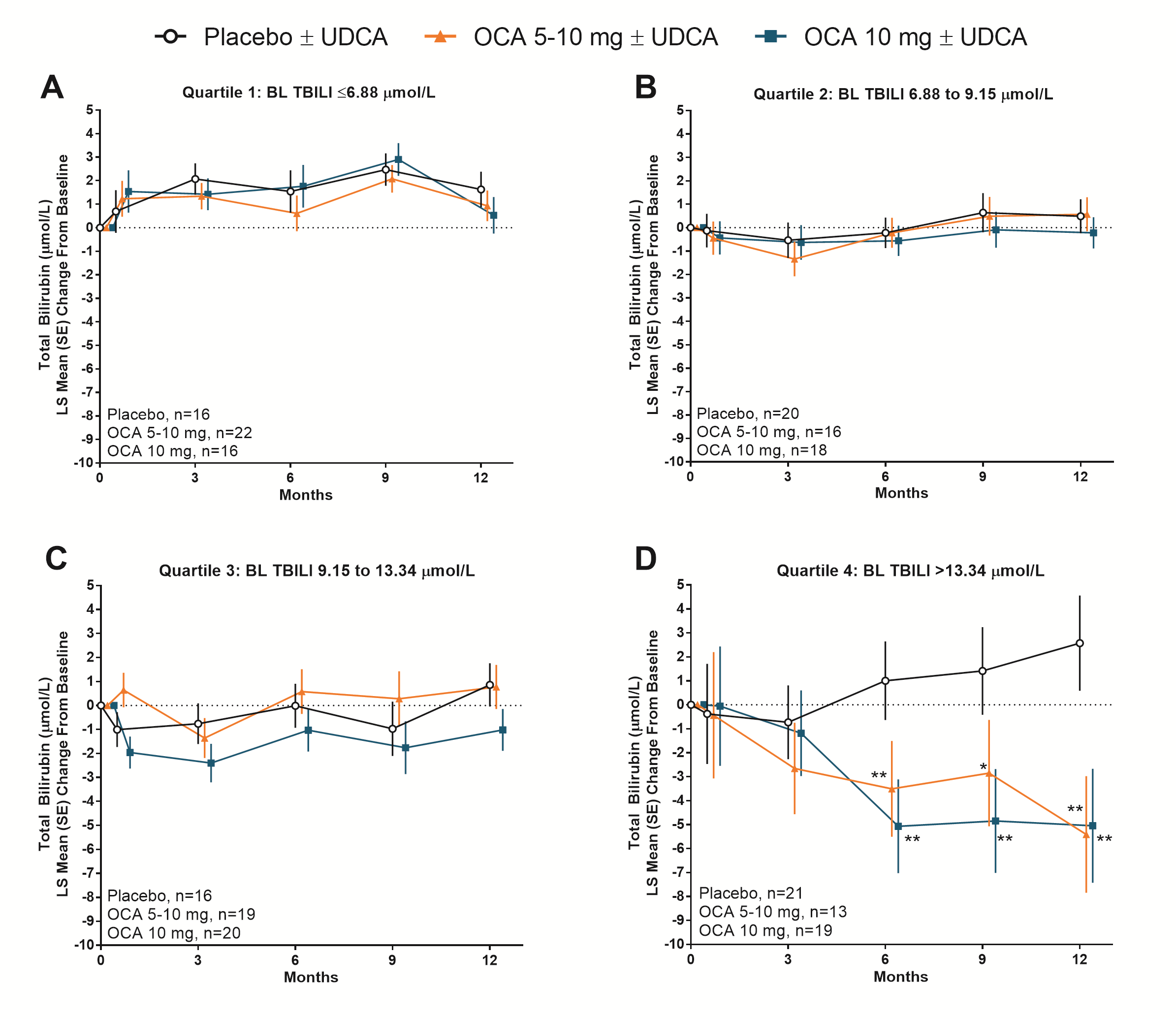
Fig. S3. Changes in total bilirubin in POISE across 12 months of treatment in each total bilirubin quartile. Patients in the POISE ITT population (N=216) were pooled across treatment groups and divided into quartiles by baseline total bilirubin levels. The ULN for total bilirubin was defined as 19.32 micromol/L for women and 25.48 micromol/L for men.

Levels of significance: *p<0.05, **p<0.01; p-values for comparing active treatments with placebo were obtained using an ANCOVA model with baseline value as a covariate and fixed effects for treatment and the randomization strata factor.

Abbreviations: ANCOVA, analysis of covariance; BL, baseline; ITT, intent-to-treat; LS, least squares; OCA, obeticholic acid; POISE, Primary biliary cholangitis OCA International Study of Efficacy; SE, standard error; TBILI, total bilirubin; UDCA, ursodeoxycholic acid; ULN, upper limit of normal.

**(QC’ed-- t355.3.3-efflabs-tbili-0.7-si-hrm-db301-15FEB2019)**


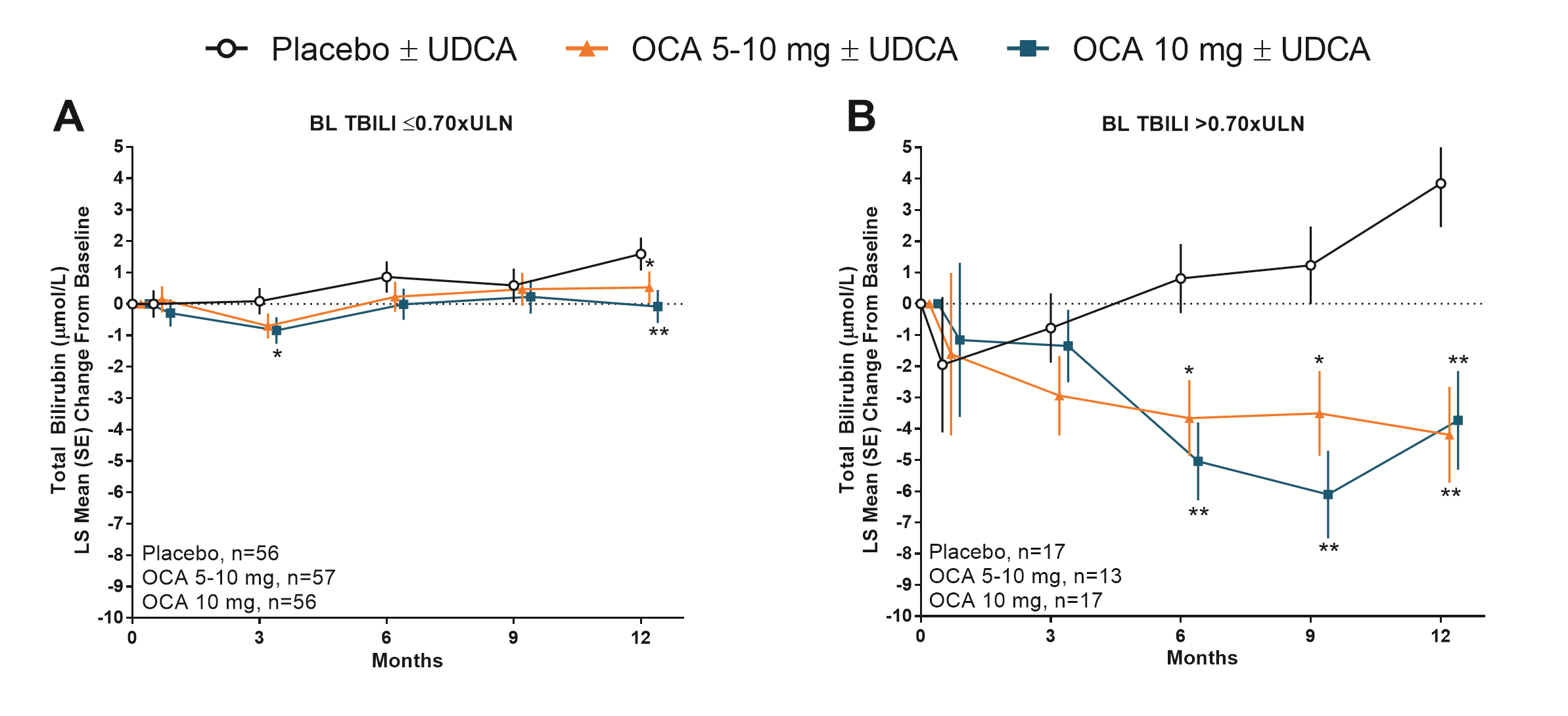
Fig. S4. Changes in total bilirubin across 12 months of treatment in the POISE double-blind phase in patients with total bilirubin ≤0.70xULN or >0.70xULN at baseline. Patients in the POISE ITT population (N=216) were divided by baseline total bilirubin levels. The ULN for total bilirubin was defined as 19.32 micromol/L for women and 25.48 micromol/L for men. (A) Patients with baseline total bilirubin levels ≤0.70xULN. (B) Patients with baseline direct bilirubin levels >0.70xULN.

Levels of significance: *p<0.05, **p<0.01; p-values for comparing active treatments with placebo were obtained using an ANCOVA model with baseline value as a covariate and fixed effects for treatment and the randomization strata factor. **(QC’ed-- Footer-- t355.3.3-efflabs-tbili-0.7-si-hrm-db301-15FEB2019)**
Abbreviations: ANCOVA, analysis of covariance; BL, baseline; ITT, intent-to-treat; LS, least squares; OCA, obeticholic acid; PBC, primary biliary cholangitis; POISE, PBC OCA International Study of Efficacy; SE, standard error; TBILI, total bilirubin; UDCA, ursodeoxycholic acid; ULN, upper limit of normal.


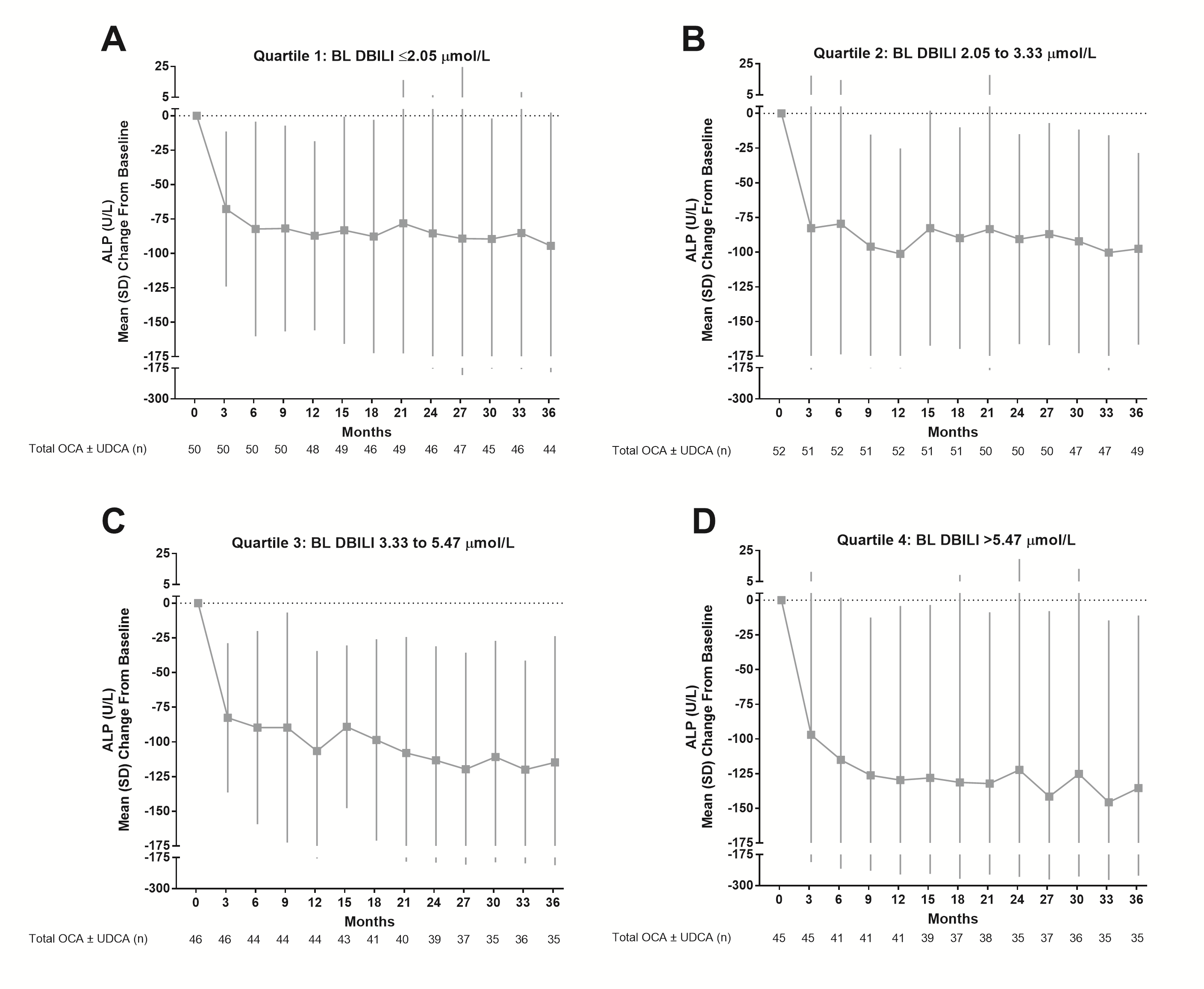
Fig. S5. Changes in ALP in the POISE OLE across 36 months of treatment in each direct bilirubin quartile by double-blind treatment in the safety population. Patients enrolled in the POISE OLE were divided into quartiles by baseline direct bilirubin levels. (A-B) Quartiles 1 and 2 represent patients with normal baseline direct bilirubin levels. (C-D) Quartiles 3 and 4 represent patients with baseline direct bilirubin levels generally above the ULN (defined as 3.42 micromol/L).

Abbreviations: ALP, alkaline phosphatase; BL, baseline; DBILI, direct bilirubin; OCA, obeticholic acid; OLE, open-label extension; PBC, primary biliary cholangitis; POISE, PBC OCA International Study of Efficacy; SD, standard deviation; UDCA, ursodeoxycholic acid; ULN, upper limit of normal.

**(QC’ed-- Pares_AASLD_2017_Bili Quartiles_slides_final, slide 6; t355.12-primeff-dbiliq-hrm-db301-13APR2017)**


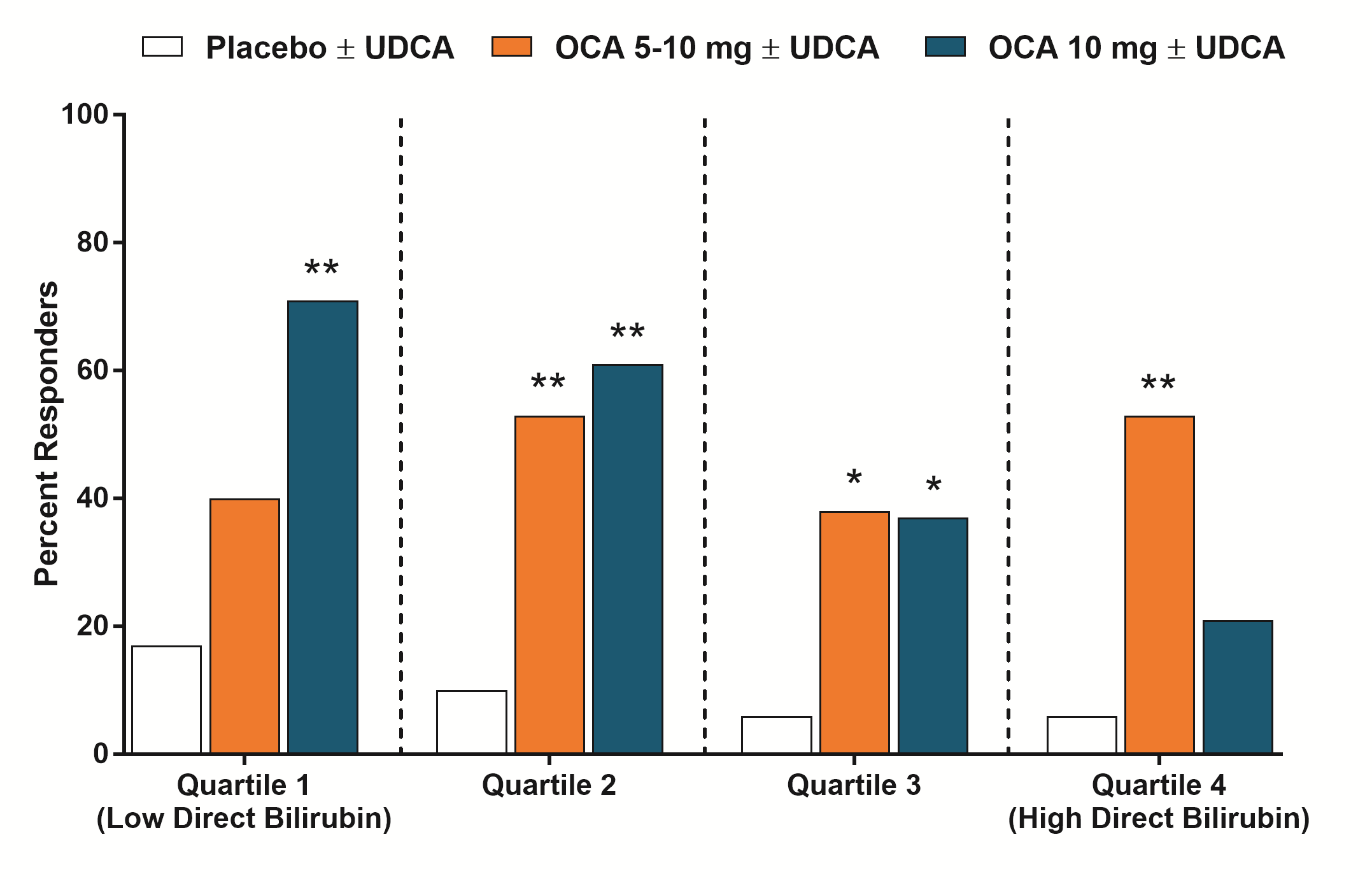


Fig. S6. Primary endpoint of POISE at 12 months of treatment in each direct bilirubin quartile in the ITT population (N=216). Patients in the POISE ITT population (N=216) were pooled across treatment groups and divided into quartiles by baseline direct bilirubin levels. Quartiles 1 and 2 represent patients with normal baseline direct bilirubin levels, and quartiles 3 and 4 represent patients with baseline direct bilirubin levels generally above the ULN (defined as 3.42 micromol/L). Responders were those who had ALP<1.67xULN with a reduction of ≥15% from baseline and total bilirubin ≤ULN at Month 12. Patient discontinuations were considered non-responders. **(Nevens, 2016, pg3; footer- t355.12-primeff-dbiliq-hrm-db301-13APR2017 )** In the fourth-quartile OCA 10-mg group, 5 patients (26%) discontinued because of pruritus. **(Pares_AASLD_2017_Bili Quartiles_Slides_Final, slide 6; footer- t355.12-primeff-dbiliq-hrm-db301-13APR2017 )**

Levels of significance: *p<0.05, **p<0.01; p-values for comparing treatments were obtained using the Cochran-Mantel-Haenszel general association test stratified by the randomization strata factor. **(footer- t355.12-primeff-dbiliq-hrm-db301-13APR2017 )**

Abbreviations: ALP, alkaline phosphatase; ITT, intent-to-treat; OCA, obeticholic acid; PBC, primary biliary cholangitis; POISE, PBC OCA International Study of Efficacy; UDCA, ursodeoxycholic acid; ULN, upper limit of normal.


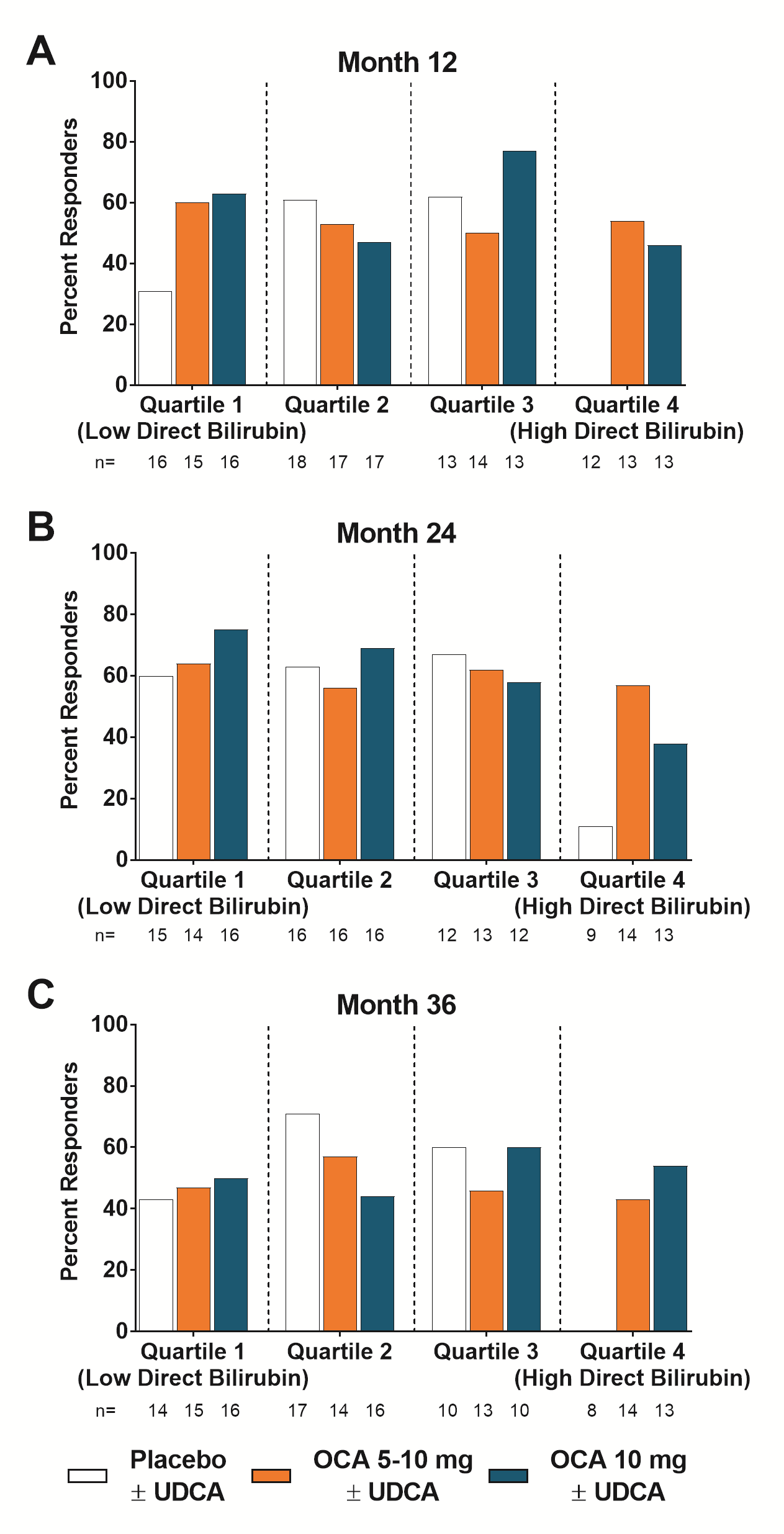
 **(QC’ed—t415 .4-primeff-dbiliq-m36-14SEP2018, starts on p 3)**

##### Fig. S7. Primary endpoint of POISE by double-blind treatment in the safety population in the OLE (N=193).

Patients enrolled in the POISE OLE were pooled across treatment groups and divided into quartiles by baseline direct bilirubin levels. Quartiles 1 and 2 represent patients with normal baseline direct bilirubin levels, and quartiles 3 and 4 represent patients with baseline direct bilirubin levels generally above the ULN (defined as 3.42 micromol/L). (A) Percent responders at 12 months of treatment in each direct bilirubin quartile. (B) Percent responders at 24 months of treatment in each direct bilirubin quartile. (C) Percent responders at 36 months of treatment in each direct bilirubin quartile. Responders were those who had ALP<1.67xULN with a reduction of ≥15% from baseline and total bilirubin ≤ULN. Patient discontinuations were considered non-responders. **(QC’ed—footer t415 .4-primeff-dbiliq-m36-14SEP2018)**

Abbreviations: ALP, alkaline phosphatase; OCA, obeticholic acid; OLE, open-label extension; PBC, primary biliary cholangitis; POISE, PBC OCA International Study of Efficacy; UDCA, ursodeoxycholic acid; ULN, upper limit of normal.

**
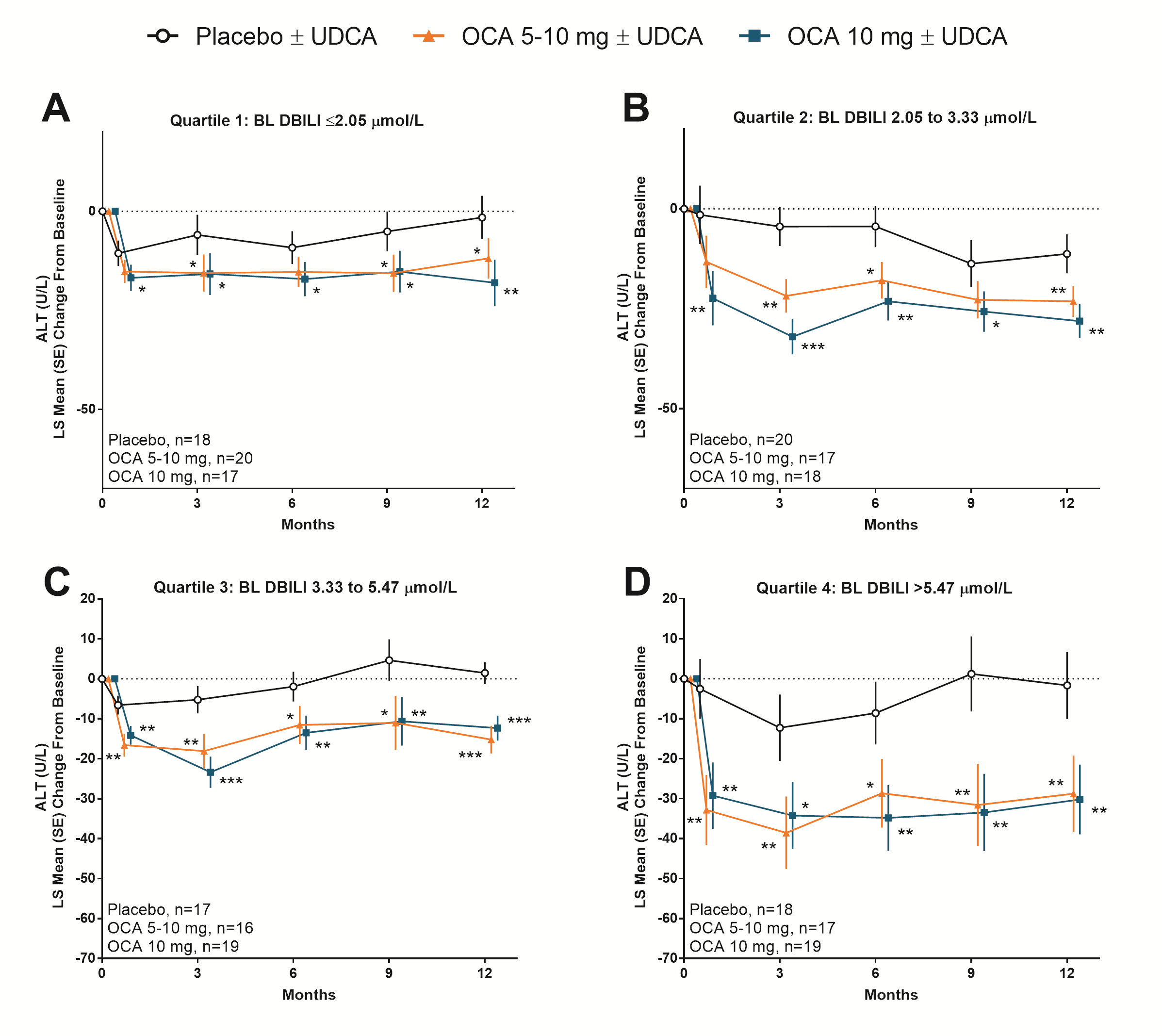
**Fig. S8. Changes in ALT in POISE across 12 months of treatment in each direct bilirubin quartile. **(t355.13.2-efflabs-dbiliq-si-hrm-db301-13APR2017_MK)**

Patients in the POISE ITT population (N=216) were pooled across treatment groups and divided into quartiles by baseline direct bilirubin levels. (A-B) Quartiles 1 and 2 represent patients with normal baseline direct bilirubin levels. (C-D) Quartiles 3 and 4 represent patients with baseline direct bilirubin levels generally above the ULN (defined as 3.42 micromol/L).

Levels of significance: *p<0.05, **p<0.01, ***p<0.0001; p-values for comparing active treatments with placebo were obtained using an ANCOVA model with baseline value as a covariate and fixed effects for treatment and the randomization strata factor.

Abbreviations: ALT, alanine aminotransferase; ANCOVA, analysis of covariance; BL, baseline; DBILI, direct bilirubin; ITT, intent-to-treat; LS, least squares; OCA, obeticholic acid; PBC, primary biliary cholangitis; POISE, PBC OCA International Study of Efficacy; SE, standard error; UDCA, ursodeoxycholic acid; ULN, upper limit of normal.

**
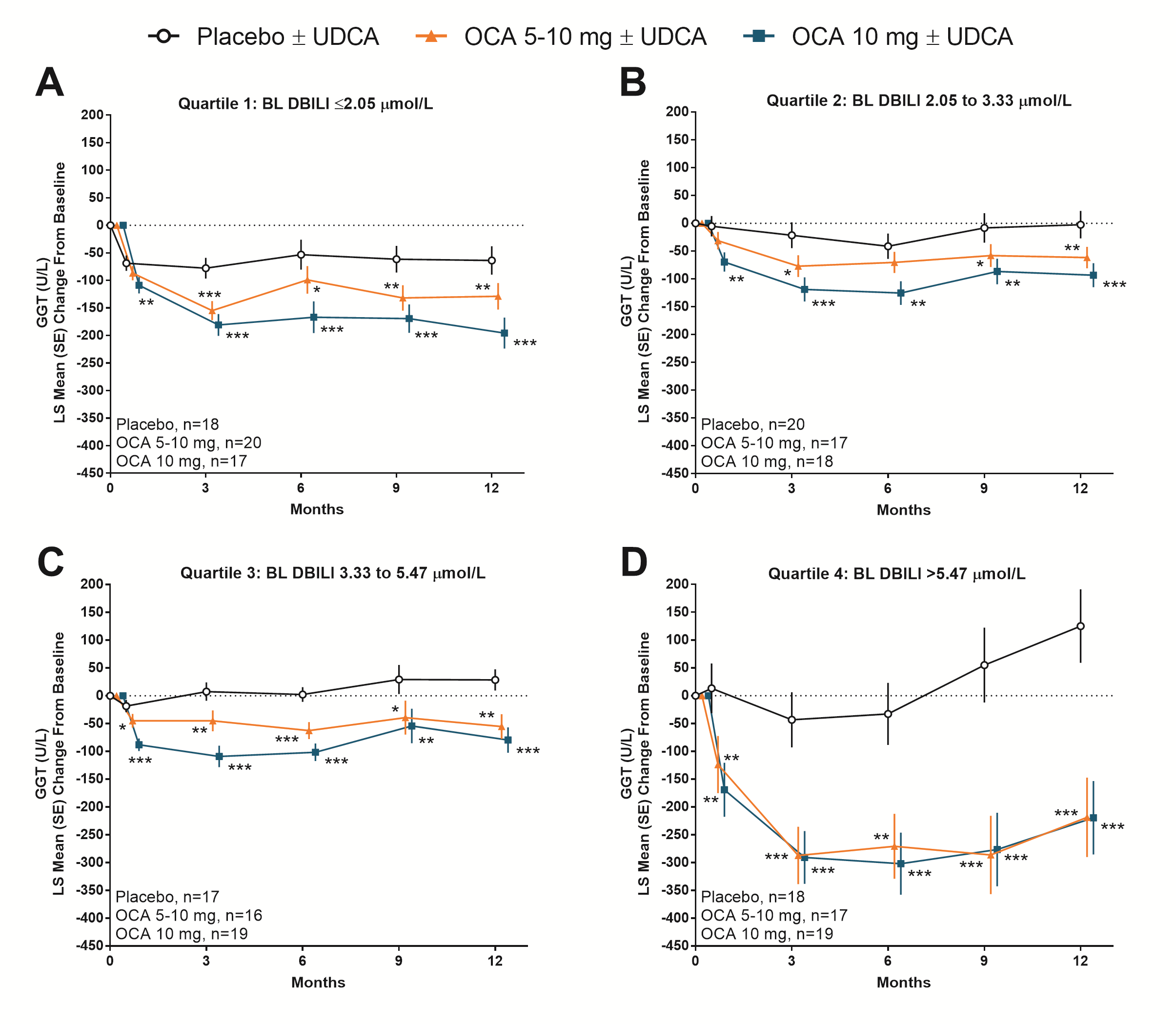
**Fig. S9. Changes in GGT in POISE across 12 months of treatment in each direct bilirubin quartile. **(t355.13.2-efflabs-dbiliq-si-hrm-db301-13APR2017_MK)**

Patients in the POISE ITT population (N=216) were pooled across treatment groups and divided into quartiles by baseline direct bilirubin levels. (A-B) Quartiles 1 and 2 represent patients with normal baseline direct bilirubin levels. (C-D) Quartiles 3 and 4 represent patients with baseline direct bilirubin levels generally above the ULN (defined as 3.42 micromol/L).

Levels of significance: *p<0.05, **p<0.01, ***p<0.0001; p-values for comparing active treatments with placebo were obtained using an ANCOVA model with baseline value as a covariate and fixed effects for treatment and the randomization strata factor.

Abbreviations: ANCOVA, analysis of covariance; BL, baseline; DBILI, direct bilirubin; GGT, gamma-glutamyl transferase; ITT, intent-to-treat; LS, least squares; OCA, obeticholic acid; PBC, primary biliary cholangitis; POISE, PBC OCA International Study of Efficacy; SE, standard error; UDCA, ursodeoxycholic acid; ULN, upper limit of normal.

**
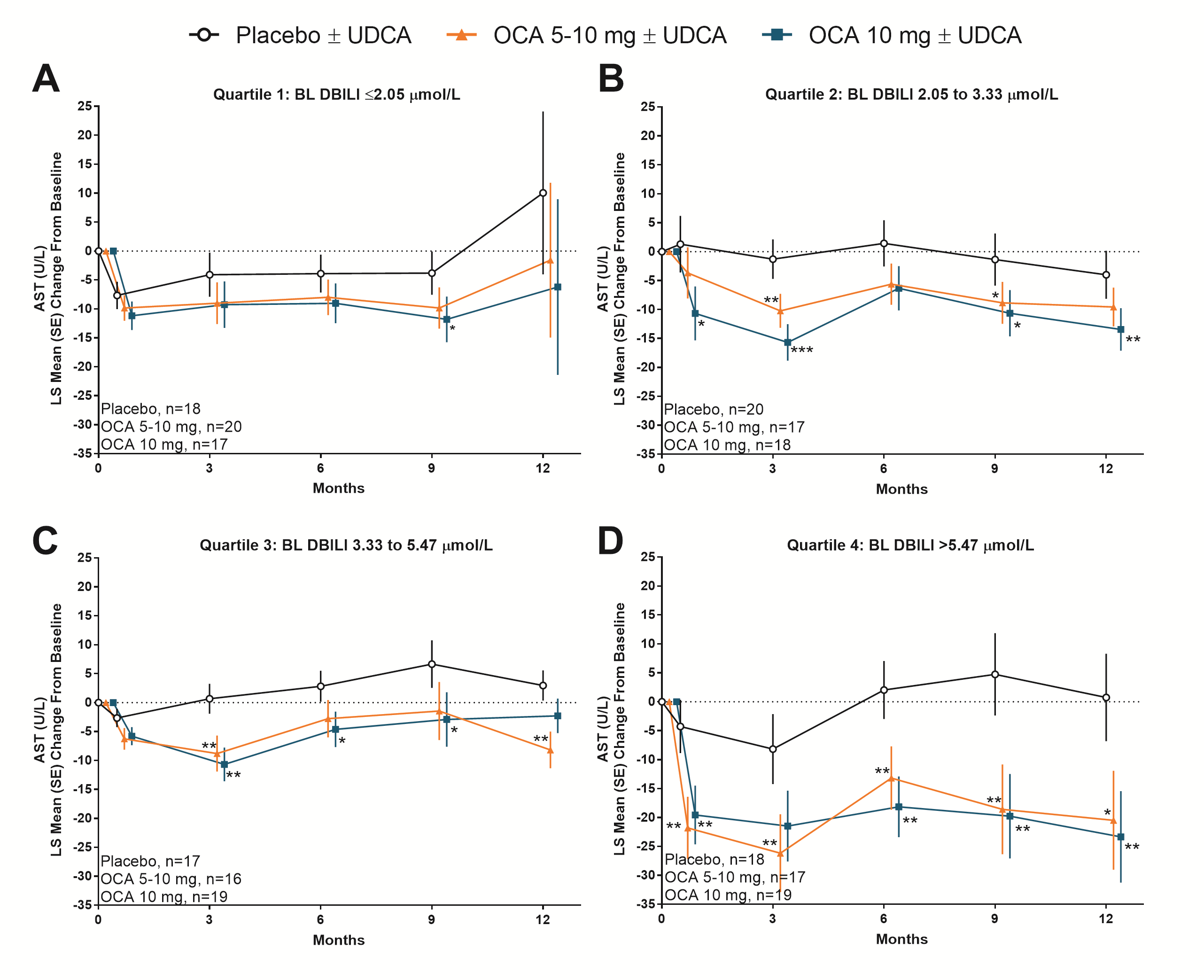
**Fig. S10. Changes in AST in POISE across 12 months of treatment in each direct bilirubin quartile. **(t355.13.2-efflabs-dbiliq-si-hrm-db301-13APR2017_MK)**

Patients in the POISE ITT population (N=216) were pooled across treatment groups and divided into quartiles by baseline direct bilirubin levels. (A-B) Quartiles 1 and 2 represent patients with normal baseline direct bilirubin levels. (C-D) Quartiles 3 and 4 represent patients with baseline direct bilirubin levels generally above the ULN (defined as 3.42 micromol/L).

Levels of significance: *p<0.05, **p<0.01, ***p<0.0001; p-values for comparing active treatments with placebo were obtained using an ANCOVA model with baseline value as a covariate and fixed effects for treatment and the randomization strata factor.

Abbreviations: ANCOVA, analysis of covariance; AST, aspartate aminotransferase; BL, baseline; DBILI, direct bilirubin; ITT, intent-to-treat; LS, least squares; OCA, obeticholic acid; PBC, primary biliary cholangitis; POISE, PBC OCA International Study of Efficacy; SE, standard error; UDCA, ursodeoxycholic acid; ULN, upper limit of normal.


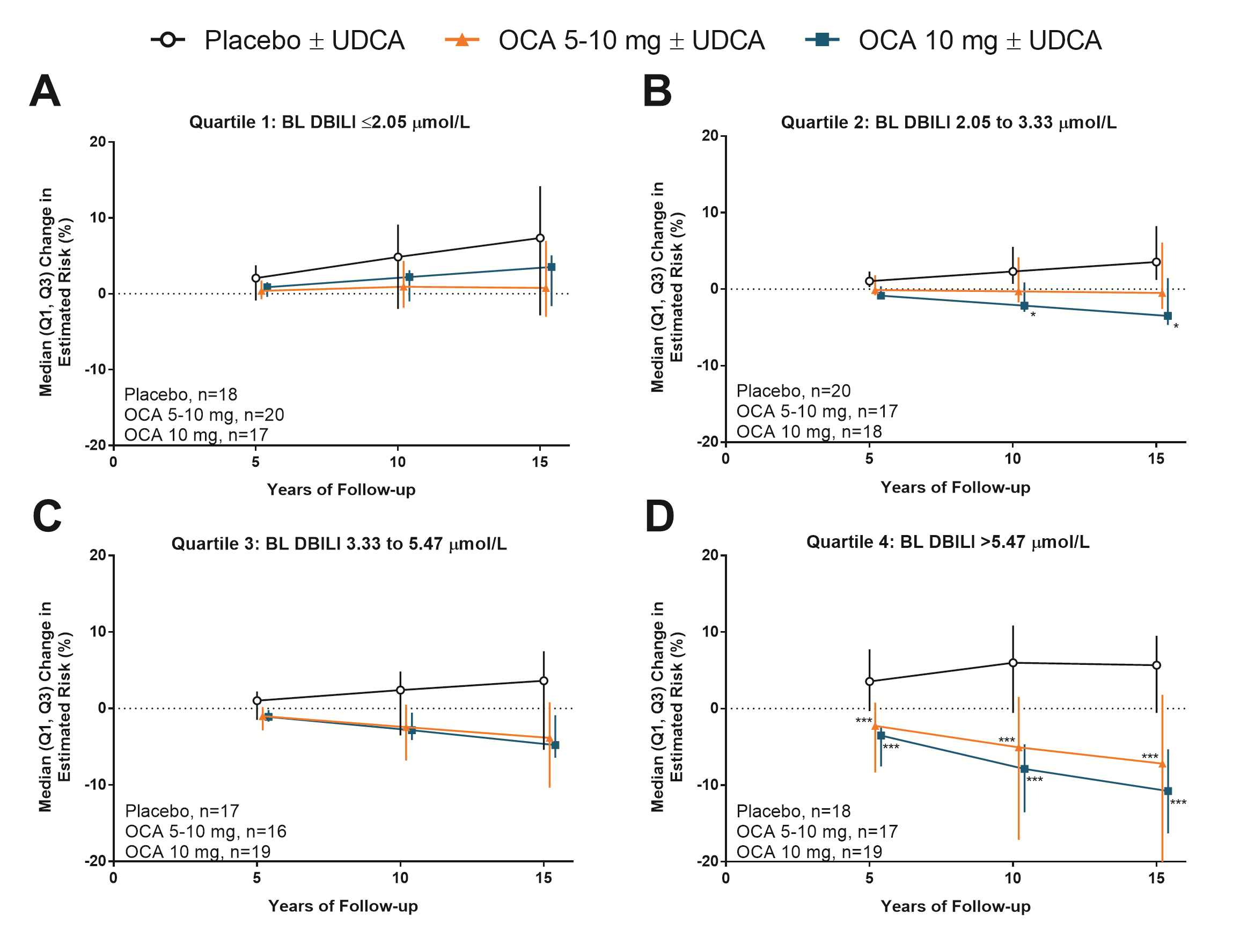
Fig. S11. Changes in estimated risk using the GLOBE score in POISE at 12 months of treatment in each direct bilirubin quartile. Patients in the POISE ITT population (N=216) were pooled across treatment groups and divided into quartiles by baseline direct bilirubin levels. (A-B) Quartiles 1 and 2 represent patients with normal baseline direct bilirubin levels. (C-D) Quartiles 3 and 4 represent patients with baseline direct bilirubin levels generally above the ULN (defined as 3.42 micromol/L).

Levels of significance: *p<0.05, ***p<0.0001; p-values for comparing active treatments with placebo were obtained using an ANCOVA model with baseline value as a covariate and fixed effects for treatment and the randomization strata factor.

Abbreviations: ANCOVA, analysis of covariance; BL, baseline; DBILI, direct bilirubin; ITT, intent-to-treat; OCA, obeticholic acid; PBC, primary biliary cholangitis; POISE, PBC OCA International Study of Efficacy; UDCA, ursodeoxycholic acid.


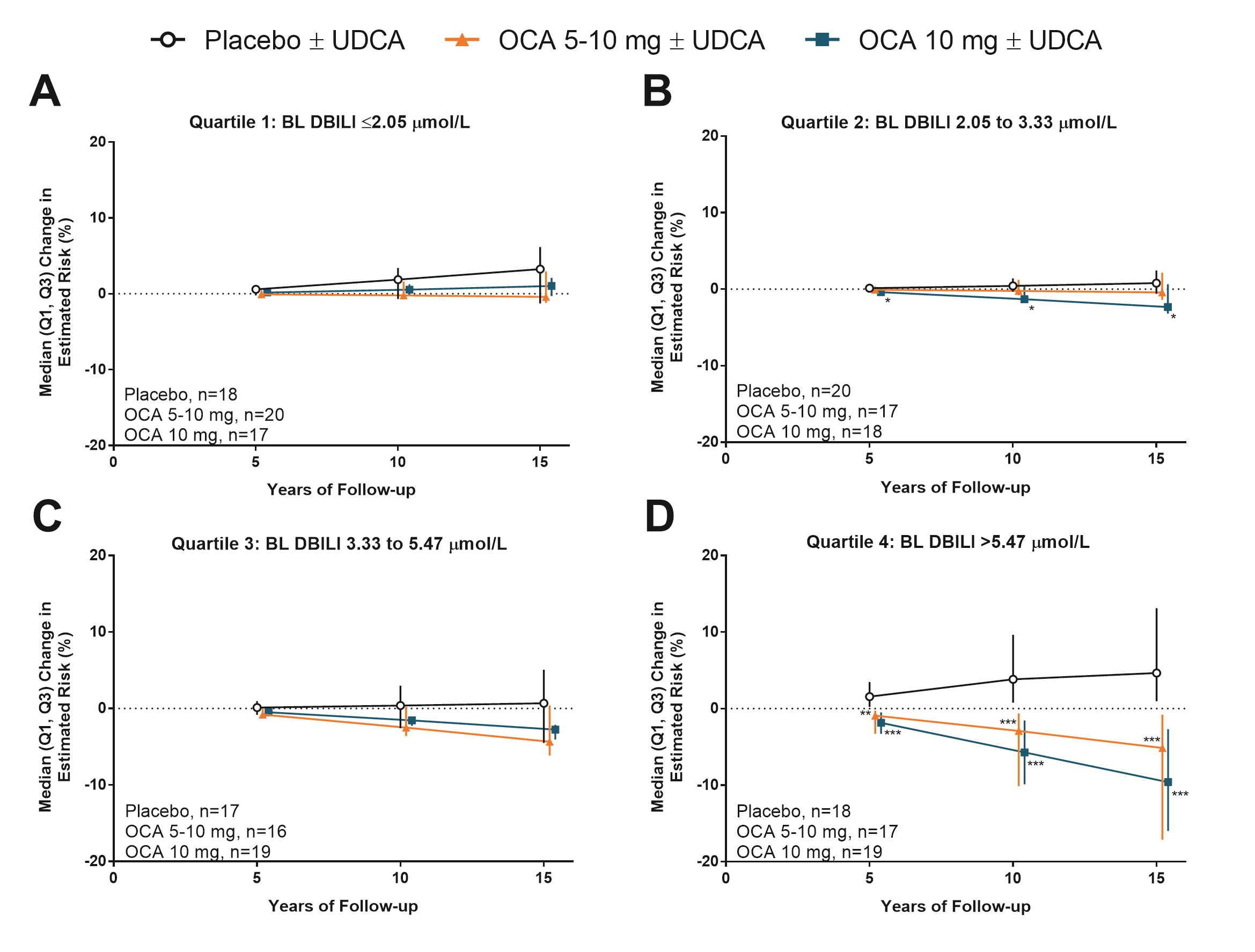
Fig. S12. Changes in estimated risk using the UK-PBC risk score in POISE at 12 months of treatment in each direct bilirubin quartile. Patients in the POISE ITT population (N=216) were pooled across treatment groups and divided into quartiles by baseline direct bilirubin levels. (A-B) Quartiles 1 and 2 represent patients with normal baseline direct bilirubin levels. (C-D) Quartiles 3 and 4 represent patients with baseline direct bilirubin levels generally above the ULN (defined as 3.42 micromol/L).

Levels of significance: *p<0.05, **p<0.01, ***p<0.0001; p-values for comparing active treatments with placebo were obtained using an ANCOVA model with baseline value as a covariate and fixed effects for treatment and the randomization strata factor.

Abbreviations: ANCOVA, analysis of covariance; BL, baseline; DBILI, direct bilirubin; ITT, intent-to-treat; OCA, obeticholic acid; PBC, primary biliary cholangitis; POISE, PBC OCA International Study of Efficacy; UDCA, ursodeoxycholic acid.

**
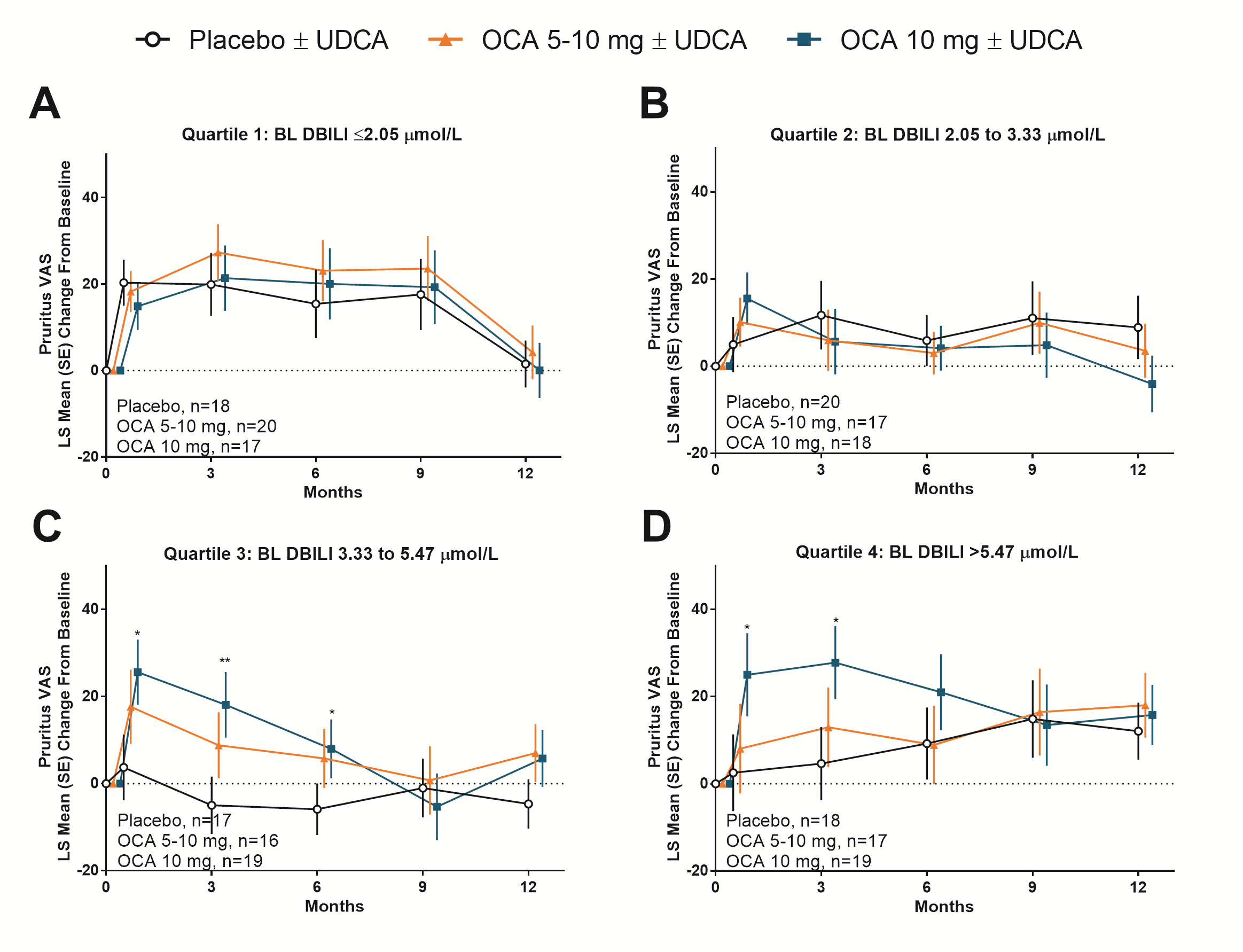
** Fig. S13. Changes in pruritus VAS scores in POISE across 12 months of treatment in each direct bilirubin quartile. **Pares_AASLD_2017_Bili Quartiles_slides_final, slide 11; t355.15-vas-dbiliq-hrm-db301-14APR2017)**

Patients in the POISE ITT population (N=216) were pooled across treatment groups and divided into quartiles by baseline direct bilirubin levels. (A-B) Quartiles 1 and 2 represent patients with normal baseline direct bilirubin levels. (C-D) Quartiles 3 and 4 represent patients with baseline direct bilirubin levels generally above the ULN (defined as 3.42 micromol/L).

Levels of significance: *p<0.05, **p<0.01; p-values for comparing active treatments with placebo were obtained using an ANCOVA model with baseline value as a covariate and fixed effects for treatment and the randomization strata factor.

Abbreviations: ANCOVA, analysis of covariance; BL, baseline; DBILI, direct bilirubin; ITT, intent-to-treat; LS, least squares; OCA, obeticholic acid; PBC, primary biliary cholangitis; POISE, PBC OCA International Study of Efficacy; SE, standard error; UDCA, ursodeoxycholic acid; ULN, upper limit of normal; VAS, visual analog scale.

**
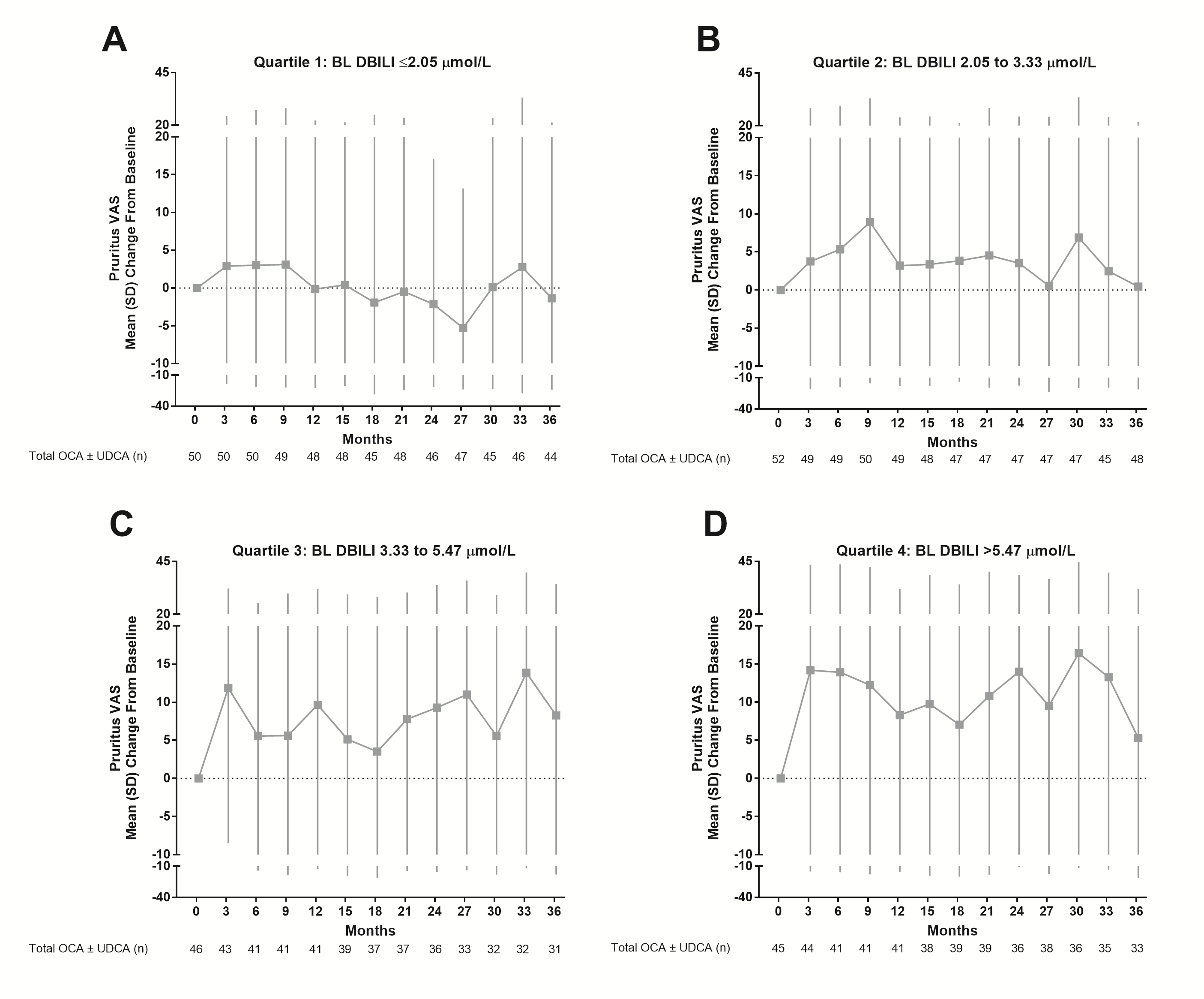
** Fig. S14. Changes in pruritus VAS scores in the POISE OLE across 36 months of treatment in each direct bilirubin quartile by double-blind treatment in the safety population. **(QC’ed-- t425.3-vas-dbiliq-oca-m36-09NOV2018)**

Patients enrolled in the POISE OLE were divided into quartiles by baseline direct bilirubin levels. (A-B) Quartiles 1 and 2 represent patients with normal baseline direct bilirubin levels. (C-D) Quartiles 3 and 4 represent patients with baseline direct bilirubin levels generally above the ULN (defined as 3.42 micromol/L).

Abbreviations: BL, baseline; DBILI, direct bilirubin; OCA, obeticholic acid; OLE, open-label extension; PBC, primary biliary cholangitis; POISE, PBC OCA International Study of Efficacy; SD, standard deviation; UDCA, ursodeoxycholic acid; ULN, upper limit of normal; VAS, visual analog scale.

Supplemental Tables Table S1. Treatment-emergent adverse events by baseline direct bilirubin quartile in POISE in the safety population. **(QC’ed-- Pares_AASLD_2017_Bili Quartiles_slides_final, slide 10; t355.14.1-ae-bysocpt-dbiliq-hrm-db301-13APR2017; t355.14.2-ae-sae-dbiliq-hrm-db301-13APR2017)**

|  | **Placebo** | **OCA 5-10 mg** | **OCA 10 mg** |
| --- | --- | --- | --- |
| **Quartile 1** | **n=18** | **n=20** | **n=17** |
| **All AEs** | 17 (94) | 18 (90) | 14 (82) |
| **Pruritus** | 9 (50) | 11 (55) | 10 (59) |
| **All SAEs** | 0 | 3 (15) | 2 (12) |
| **Quartile 2** | **n=20** | **n=17** | **n=18** |
| **All AEs** | 18 (90) | 15 (88) | 18 (100) |
| **Pruritus** | 11 (55) | 10 (59) | 10 (56) |
| **All SAEs** | 1 (5) | 3 (18) | 2 (11) |
| **Quartile 3** | **n=17** | **n=16** | **n=19** |
| **All AEs** | 16 (94) | 15 (94) | 19 (100) |
| **Pruritus** | 1 (6) | 8 (50) | 14 (74) |
| **All SAEs** | 1 (6) | 1 (6) | 1 (5) |
| **Quartile 4** | **n=18** | **n=17** | **n=19** |
| **All AEs** | 15 (83) | 17 (100) | 18 (95) |
| **Pruritus** | 7 (39) | 10 (59) | 16 (84) |
| **All SAEs** | 1 (6) | 4 (24) | 3 (16) |

Data are n (%).

Quartiles were defined by baseline direct bilirubin levels as follows: quartile 1, ≤2.05 µmol/L; quartile 2, 2.05 to 3.33 µmol/L; quartile 3, 3.33 to 5.47 µmol/L; quartile 4, >5.47 µmol/L.

A treatment-emergent adverse event is any adverse event that newly appeared, increased in frequency, or worsened in severity after the initiation of OCA.

Abbreviations: AE, adverse event; OCA, obeticholic acid; PBC, primary biliary cholangitis; POISE, PBC OCA International Study of Efficacy; SAE, serious adverse event.

**Table S2. Exposure-adjusted treatment-emergent adverse event rate by baseline direct bilirubin quartile in the POISE OLE in the safety population. (Table provided by Intercept and QC’ed by Intercept but doubled checked by JB;** **t415.6-sae-soc-pt-exp-adj-dbiliq-m36-20DEC2018; t415.5-ae-soc-pt-exp-adj-dbiliq-m36-19DEC2018)**

|  | **Total OCA** |
| --- | --- |
| **Quartile 1** | **n=50** |
| **All AEs** | 191.40 |
| **Pruritus** | 27.90 |
| **All SAEs** | 7.10 |
| **Hepatobiliary Disorders** | <1 |
| **Quartile 2** | **n=52** |
| **All AEs** | 241.70 |
| **Pruritus** | 31.80 |
| **All SAEs** | 6.50 |
| **Hepatobiliary Disorders** | <1 |
| **Quartile 3** | **n=46** |
| **All AEs** | 431.80 |
| **Pruritus** | 56.50 |
| **All SAEs** | 10.80 |
| **Hepatobiliary Disorders** | <1 |
| **Quartile 4** | **n=45** |
| **All AEs** | 442.30 |
| **Pruritus** | 68.90 |
| **All SAEs** | 17.30 |
| **Hepatobiliary Disorders** | 2.20 |

Quartiles were defined by baseline direct bilirubin levels as follows: quartile 1, ≤2.05 µmol/L; quartile 2, 2.05 to 3.33 µmol/L; quartile 3, 3.33 to 5.47 µmol/L; quartile 4, >5.47 µmol/L.

A treatment-emergent adverse event is any adverse event that newly appeared, increased in frequency, or worsened in severity after initiation of OCA in the OLE phase. Exposure-adjusted incidence is the number of unique patients experiencing the given treatment-emergent adverse event/total patient exposure years*100. An individual’s patient exposure years is calculated as (first treatment-emergent adverse event start date ‒ first OCA dose date in the OLE + 1)/365.25 for patients who experienced the given AE and (last OCA dose date ‒ first OCA dose date in the OLE + 1)/365.25 for patients who did not experience the given AE. **Footer-- t415.5-ae-soc-pt-exp-adj-dbiliq-m36-19DEC2018**

Abbreviations: AE, adverse event; OCA, obeticholic acid; OLE, open-label extension; POISE, Primary biliary cholangitis OCA International Study of Efficacy; SAE, serious adverse event.
